# Diagnosis of 25 genotypes of human papillomaviruses for their physical statuses in cervical precancerous/cancerous lesions: a comparison of E2/E6E7 ratio-based vs. multiple E1-L1/E6E7 ratio-based detection techniques

**DOI:** 10.1186/s12967-014-0282-2

**Published:** 2014-10-02

**Authors:** Rong Zhang, Yi-feng He, Mo Chen, Chun-mei Chen, Qiu-jing Zhu, Huan Lu, Zhen-hong Wei, Fang Li, Xiao-xin Zhang, Cong-jian Xu, Long Yu

**Affiliations:** Department of Obstetrics and Gynecology, Fengxian Hospital, Southern Medical University, 6600 Nanfeng Road, Shanghai, 201499 China; Department of Obstetrics and Gynecology, Ren Ji Hospital, School of Medicine, Shanghai Jiao Tong University, 160 Pujian Road, Shanghai, 200127 China; Shanghai Key Laboratory of Gynecologic Oncology, Ren Ji Hospital, School of Medicine, Shanghai Jiao Tong University, 160 Pujian Road, Shanghai, 200127 China; Department of Gynecology, Obstetrics and Gynecology Hospital, Fudan University, 419 Fangxie Road, Shanghai, 200011 China; State Key Laboratory of Genetic Engineering, Institute of Genetics, School of Life Sciences, Fudan University, 220 Handan Road, Shanghai, 200433 China

**Keywords:** Human papillomavirus, Physical status, Integration, E1, E2, E6, E7, L1, Polymerase chain reaction, Cervical cancer

## Abstract

**Background:**

Cervical lesions caused by integrated human papillomavirus (HPV) infection are highly dangerous because they can quickly develop into invasive cancers. However, clinicians are currently hampered by the lack of a quick, convenient and precise technique to detect integrated/mixed infections of various genotypes of HPVs in the cervix. This study aimed to develop a practical tool to determine the physical status of different HPVs and evaluate its clinical significance.

**Methods:**

The target population comprised 1162 women with an HPV infection history of > six months and an abnormal cervical cytological finding. The multiple E1-L1/E6E7 ratio analysis, a novel technique, was developed based on determining the ratios of E1/E6E7, E2/E6E7, E4E5/E6E7, L2/E6E7 and L1/E6E7 within the viral genome. Any imbalanced ratios indicate integration. Its diagnostic and predictive performances were compared with those of E2/E6E7 ratio analysis. The detection accuracy of both techniques was evaluated using the gold-standard technique “detection of integrated papillomavirus sequences” (DIPS). To realize a multigenotypic detection goal, a primer and probe library was established.

**Results:**

The integration rate of a particular genotype of HPV was correlated with its tumorigenic potential and women with higher lesion grades often carried lower viral loads. The E1-L1/E6E7 ratio analysis achieved 92.7% sensitivity and 99.0% specificity in detecting HPV integration, while the E2/E6E7 ratio analysis showed a much lower sensitivity (75.6%) and a similar specificity (99.3%). Interference due to episomal copies was observed in both techniques, leading to false-negative results. However, some positive results of E1-L1/E6E7 ratio analysis were missed by DIPS due to its stochastic detection nature. The E1-L1/E6E7 ratio analysis is more efficient than E2/E6E7 ratio analysis and DIPS in predicting precancerous/cancerous lesions, in which both positive predictive values (36.7%-82.3%) and negative predictive values (75.9%-100%) were highest (based on the results of three rounds of biopsies).

**Conclusions:**

The multiple E1-L1/E6E7 ratio analysis is more sensitive and predictive than E2/E6E7 ratio analysis as a triage test for detecting HPV integration. It can effectively narrow the range of candidates for colposcopic examination and cervical biopsy, thereby lowering the expense of cervical cancer prevention.

**Electronic supplementary material:**

The online version of this article (doi:10.1186/s12967-014-0282-2) contains supplementary material, which is available to authorized users.

## Background

**Figure 1:**
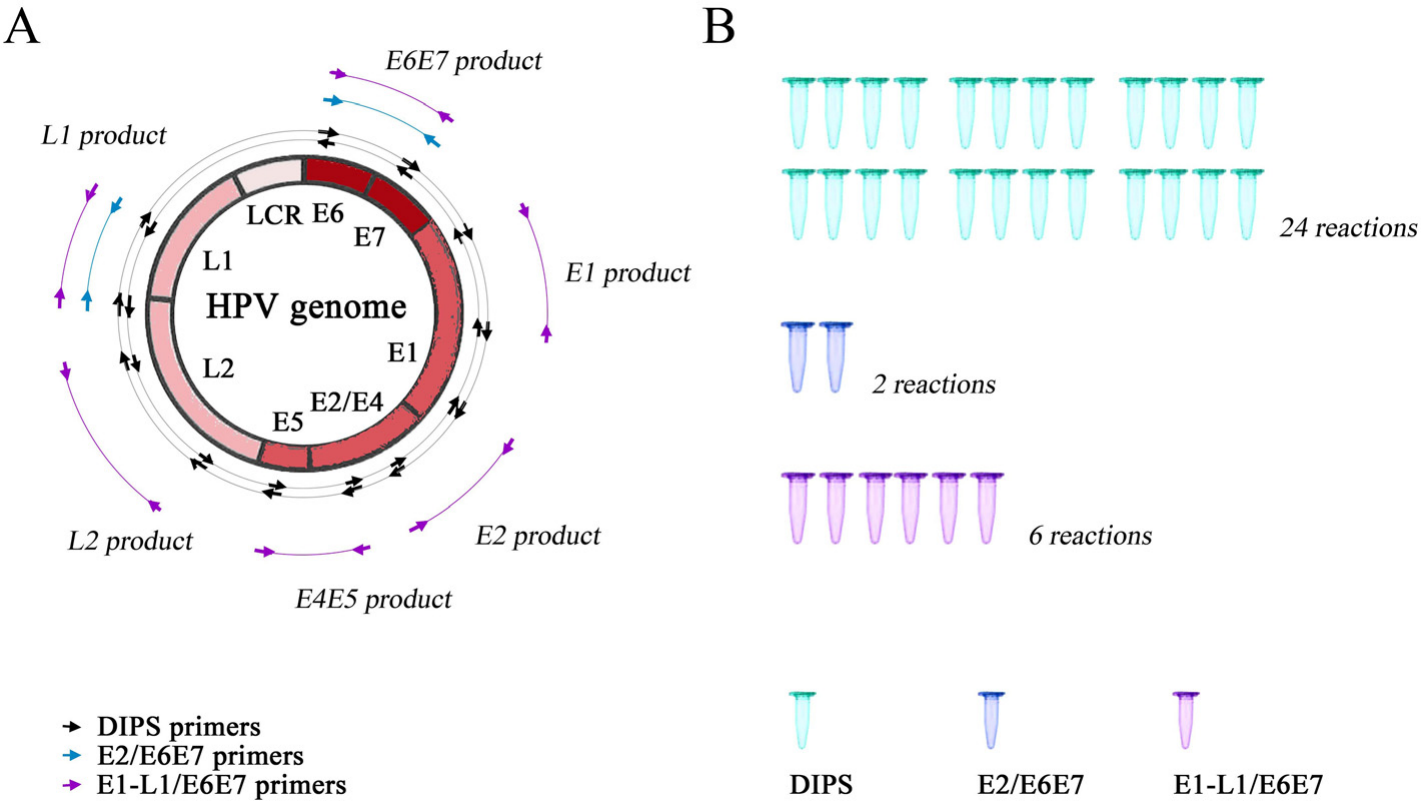
**Schematic representation of the primer design strategy and required DNA amplification reactions. A**. For the E1-L1/E6E7 ratio analysis, six pairs of primers, corresponding to the gene regions E1, E2, E4E5, L2, L1 and E6E7, were evenly arranged along the genome sequence of HPV. The products of each primer pair were 1–2 kb in length. Of these pairs, the E2 and E6E7 primer pairs were used for the E2/E6E7 ratio analysis. All of the 12 component primers and their complementary sequences were also used in the DIPS-PCR. (refer to Additional file 2: Figure S1). **B**. The numbers of DNA amplification reactions respectively required by each detection technique are displayed.

Human papillomavirus (HPV) is a sexually transmitted pathogen that has been linked to more than 90% of cervical cancer events in women of childbearing age [1]. The genome of this virus is composed of a covalently closed circular DNA molecule, of which the viral gene components are arranged in the order of DNA replication origin (ori)-long control region (LCR)-E6-E7-E1-E2-E4-E5-L2-L1 (note: “E” refers to “early gene”; “L” refers to “late gene”; see Figure 1A) [2]. The natural course for an initial HPV infection to develop into a precancerous/cancerous lesion in the uterine cervix usually takes a long period, even a decade [3]. Persistent infection with HPV, therefore, is regarded as a prerequisite to the generation of cervical cancer [1-3]. Given these conditions, detection techniques that can accurately diagnose persistent HPV infection when there are no visible precancerous/cancerous changes in the cervix are demanded by clinicians.

HPV genomic integration is a form of persistent viral infection [4]. A series of large-scale population-based epidemiological studies have demonstrated that integrated HPV can persist much longer than can non-integrated HPV (i.e., episomal HPV) [4-10]. In addition, epidemiological investigations have reported that the integrated infection rate reached 90%-100% in HPV-positive cervical epithelium, which eventually develops into the canceration stage [4-7]. The role of HPV integration in the infection-persistence mechanism could be attributed, to a greater degree, to the host cells losing their ability to discriminate the pathogenic (i.e., HPV) DNA from their own and, therefore, the ability of these cells to eliminate the pathogenic DNA and, to a lesser degree, to the viral DNA being covalently linked into host chromosomes and being spared a fate of being diluted by cell division [8]. However, viral integration impairs the genomic stability of the host cells. Chromosomal abnormalities are frequently found at virus insertion sites, and the neighboring oncogenes or tumor suppressor genes could thus be activated or repressed [9,10].

Previous in vitro experiments have confirmed that in precancerous cervical epithelial cells that originally harbored only episomal HPV copies, the frequency of HPV integration into the human genome increases with cell division and with the canceration degree that these cells exhibit [11]. Therefore, a test of the physical status of HPV DNA could be more effective than are traditional methods that simply measure viral copies to assess the risk of cervical cancer. However, so far, there are few detection tools available for performing a routine examination for integrated HPV infection in the clinical setting. Most of the existing techniques for detecting HPV integration, such as the “detection of integrated papillomavirus sequences (DIPS)” [12] and the “amplification of papilloma virus oncogene transcripts (APOT)” [13], rely on a precise DNA sequencing-based analysis and are extremely complex in their operation protocols, making these techniques both labor-intensive and time-consuming.

In this study, we developed a novel multiple E1-L1/E6E7 gene copy number ratio-based technique (henceforth referred to as “multiple E1-L1/E6E7 ratio analysis”) to detect integrated HPV infection in the cervix. This technique is derived from an already existing but less sensitive E2/E6E7 gene copy number ratio-based polymerase chain reaction (PCR) technique (henceforth referred to as “E2/E6E7 ratio analysis”) [14,15]. To improve its performance in discerning the HPV physical status, we extended the original 1-kb PCR-amplification region within the HPV E2 gene to a 6-kb region that contains viral genes from E1 through L1. The principle of the copy number ratio-based technique is that HPV integration often occurs within the E1-L1 gene regions, especially the E2 region, and if the viral DNA disruption site occurs within these regions, the corresponding PCR products should be diminished [15]. Our aim was to determine whether the extended PCR detection region is helpful to enhance the efficacy of the copy number ratio-based method for detecting the integrated infection of HPV and whether this novel technique can be used as a triage test to screen for dangerous precancerous/cancerous cases in women with a positive cervical HPV DNA test and abnormal cytological examination findings.

## Methods

### Study population

From January 1, 2011 through December 31, 2012, cervical cytological samples that were diagnosed with pathological abnormalities (e.g., atypical squamous/glandular cells, squamous intraepithelial lesions, etc., using the terminology of the 2001 Bethesda system [16]) in Pap smear or liquid-based cytological examinations (ThinPrep 2000, Hologic, Bedford, Massachusetts, USA) were collected from patients who accepted the colposcopy-based cervical biopsy in Fengxian Hospital, Southern Medical University; Obstetrics and Gynecology Hospital, Fudan University; and Renji Hospital, Jiaotong University in Shanghai, China. Informed consent forms were signed by all of the enrolled women, and the biopsy samples were independently reviewed by two pathologists through hematoxylin and eosin (H & E)-stained sections. As an inclusion criterion, all of the women were required to persistently test positive in their cervical HPV DNA tests (Multiple-genotype HPV Fluorescent Quantitative PCR Detection Kit, Fosun, Shanghai, China) for at least 6 months before the biopsy. After the first biopsy, the women who had no precancerous or cancerous changes found were requested to repeat an cervical biopsy one month later, and if the biopsy result was negative again, the women were required to repeat the course for a third time. The women who had three normal/cervicitis results were considered negative cases. The research protocol was approved by the ethics committee of Fengxian District Central Hospital.

### HPV genotyping

For each patient, the total DNA was isolated from a 15-mL cervical cytological sample (collected by cervical brushing) using a QIAamp DNA Mini Kit (Qiagen, Shenzhen, Guangdong, China) and maintained in phosphate-buffered saline at −20°C). The quality of the DNA samples was tested by measuring the β-actin (ACTB) copies. An ACTB primer pair (Additional file 1: Table S1) and a corresponding TaqMan probe (Additional file 1: Table S1) were used. The synthesized ACTB DNA template (note: all of the primers, probes and templates that were used in this study were synthesized by Sangon, Shanghai, China) was used to construct the standard curve for the real-time PCR system. Only those samples containing the ACTB copy content greater than 1 × 10^−8^ μM (equal to 10^3^ cells/μL) were considered qualified samples. The consensus HPV L1 primer pair MY09/11 was adopted to amplify viral genomic DNA. The resultant PCR product was hybridized with a panel of 45 HPV-genotypic probes on a positively charged nylon membrane (GE, Piscataway, New Jersey, USA) based on the method described by Oh et al. [16]. The patients with a multigenotypic HPV infection were excluded from the study population.

### HPV DNA load analysis

For each HPV genotype, a pair of E6E7-specific primers (Additional file 1: Table S1) and a corresponding TaqMan probe (Additional file 1: Table S1) were used. The synthesized viral genotype-specific E6E7 DNA templates were adjusted to 1 × 10^−3^ μM, and their 1/10 serial dilutions were used to construct the standard curves for the real-time PCR system. The mean E6E7/ACTB ratio was calculated based on three replicates of E6E7 and ACTB quantitative tests in each cervical sample. The viral DNA load was represented as the per cell E6E7 copy number (note: each human cell contains two copies of ACTB gene in general).

### E2/E6E7 ratio analysis

Based on the viral genotyping results, the genotype-specific E2 primer pairs and E6E7 primer pairs (Additional file 1: Table S1) were used for the real-time PCR-based E2/E6E7 ratio analysis (Figure 1A). The TaqMan probes targeting the E2 and E6E7 gene regions were used in the real-time PCR, and their detailed sequences are listed in Additional file 1: Table S1. For each viral genotype, the E2 and E6E7 DNA templates were synthesized and adjusted to 1 × 10^−3^ μM; 1/10 serial dilutions of the two templates were used to construct the standard curves. The E2/E6E7 ratio was used to evaluate the physical status of HPV. The ratios that were greater than 0.90, between 0.1 and 0.90, and less than 0.1 were considered episomal, mixed and integrated HPV infections, respectively.

### Multiple E1-L1/E6E7 ratio analysis

For the multiple E1-L1/E6E7 ratio analysis (Figure 1A), the appropriate E1, E2, E4E5, L2 and L1 primer pairs, E6E7 primer pair and corresponding TaqMan probes were used (Additional file 1: Table S1). The E1, E2, E4E5, L2, L1 and E6E7 DNA templates were synthesized for each HPV genotype and adjusted to 1 × 10^−3^ μM, and 1/10 serial dilutions of these templates were used in the standard curve constructions. To establish the viral physical status, the E1/E6E7, E2/E6E7, E4E5/E6E7, L2/E6E7 and L1/E6E7 ratios were divided into three classes. Class 1 included all of the ratios that were greater than 0.90, indicating episomal HPV infection; Class 2 included the ratios between 0.1 and 0.90, indicating mixed HPV infection; and Class 3 included the ratios less than 0.1, indicating integrated HPV infection.

### DIPS

The DIPS analysis was performed based on the methods of Luft et al. [12] with necessary modifications for adapting different HPV genotypes. Briefly, the cellular genomic DNA (0.6 mg) was digested with TaqI or Sau3AI (10 units, New England Biolabs, Ipswich, Massachusetts, USA). Enzyme-specific adapters were ligated to the digested DNA using the T4-DNA ligase (10 units, New England Biolabs). The obtained adapter-ligated DNA fragments were amplified through a two-step PCR protocol. In the initial PCR step, the DNA fragments were linearly amplified using a single HPV genotype-specific viral primer, and then in the second step, the obtained DNA product was further exponentially amplified using a HPV genotype-specific viral primer and an adapter-specific primer (Additional file 1: Table S1). For each sample, the 12 HPV genotype-specific viral primers and their reversely complementary counterparts were seriatim used in combination with the adapter-specific primer to amplify the viral-cellular junction-containing sequence (Figure 1A). The PCR products that were obtained from any primer pair of the above 24 combinations were sequenced to check the viral-cellular junction. The Caski, SiHa, and HeLa cell lines were used as positive controls.

### Statistical analysis

The two-sided Student’s t test was used to compare the means of age, parity and gravidity between the enrolled and pre-excluded women. A one-way ANOVA was used to compare the means of viral DNA loads between women with different grades of cervical lesions. The two-sided χ2 test was used to compare categorical data, such as differences in the cyto-pathological findings between the enrolled and pre-excluded women, differences in the age-related, genotype-related and cervical lesion-related distribution patterns between integrated/mixed infection and episomal HPV infection and differences in the numbers of false-positive cases and false-negative cases as detected by the E2/E6E7 ratio analysis or E1-L1/E6E7 ratio analysis among the viral load tertiles. Pearson’s product–moment correlation coefficient was used to estimate the relationship between the integration rates and tumorigenic rates of the 25 HPV genotypes. The κ value was used to estimate the degree of consistency between the cases that were diagnosed by the E2/E6E7 ratio analysis, E1-L1/E6E7 ratio analysis and DIPS analysis. The SPSS 17.0 software package (IBM, Armonk, New York, USA) was used, and p < 0.05 was defined as statistically significant.

## Results

**Figure 2:**
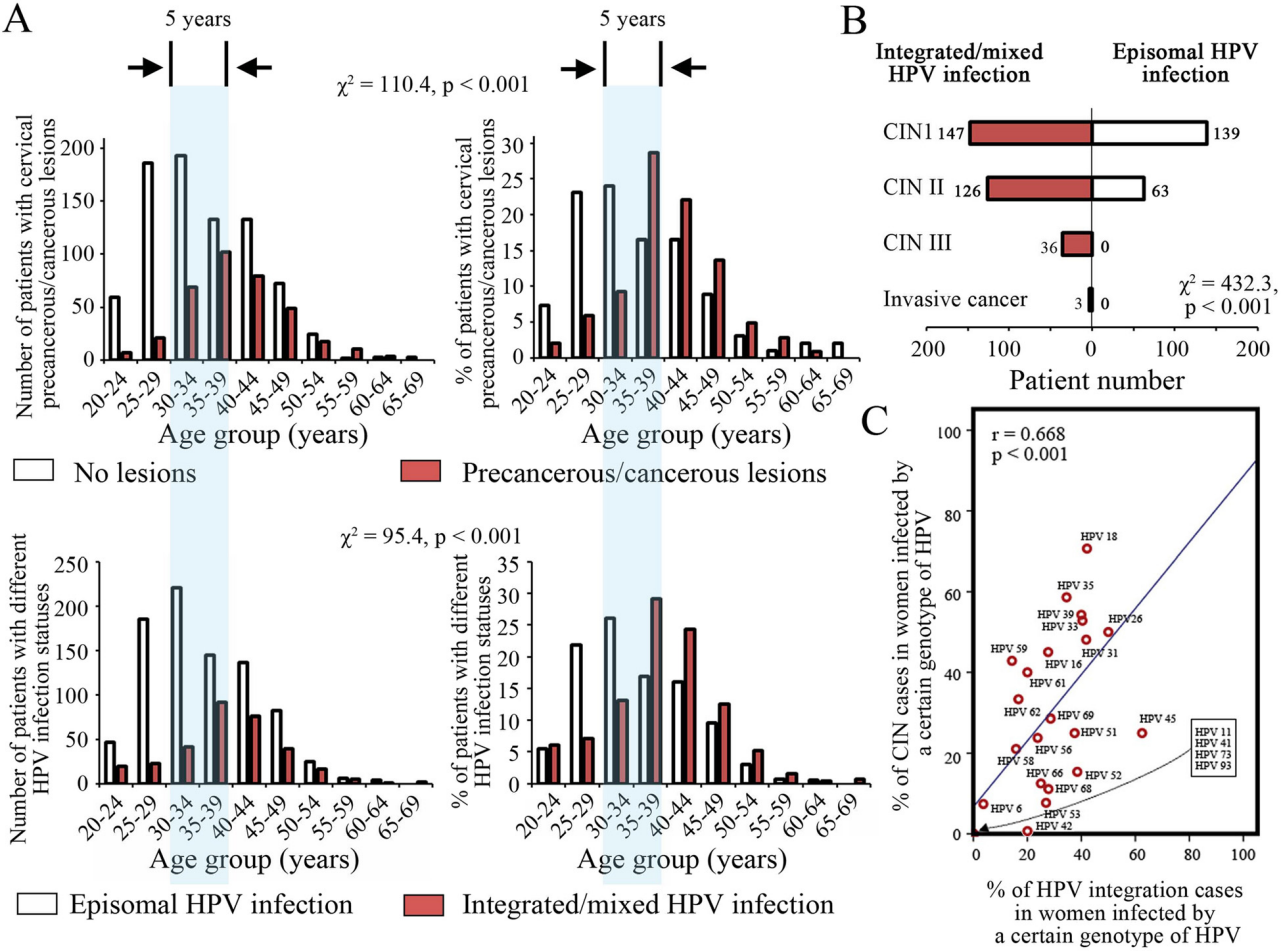
**The relationship between cervical lesions and HPV integration. A**. The age-related distribution patterns of cervical lesions (upper panel) and HPV integration events (lower panel, detected with DIPS) in the enrolled women. A significant time retardation was observed between women with cervical precancerous/cancerous lesions and those without lesions as well as between women with cervical integrated/mixed HPV infections and those with episomal infections. **B**. The cervical lesion-related distribution pattern of HPV integration is displayed. **C**. The relationship between the HPV integration rate and its tumorigenic rates was analyzed using the Pearson’s product–moment correlation coefficient, r. Blue line, the linear fitting curve.

A total of 1162 women with single-genotypic HPV infections in the cervix were enrolled. All of the cervical biopsies were performed due to abnormal findings in the cervical cytological examinations (Table 1). The mean age was 36.1 years (range: 21–68 years), and the age-related distribution pattern is shown in Figure 2A. The HPV L1 genotyping results revealed 25 viral genotypes, namely, (from high to low in the order of case number) HPV 16, 18, 33, 31, 39, 35, 6, 53, 56, 58, 68, 66, 52, 11, 51, 45, 59, 69, 62, 61, 42, 41, 26, 74 and 93. The corresponding numbers (percentages) were 602 (51.8%), 126 (10.8%), 89 (7.7%), 79 (6.8%), 35 (3.0%), 29 (2.5%), 27 (2.3%), 26 (2.2%), 21 (1.8%), 19 (1.6%), 18 (1.5%), 16 (1.4%), 13 (1.1%), 9 (0.8%), 8 (0.7%), 8 (0.7%), 7 (0.6%), 7 (0.6%), 6 (0.5%), 5 (0.4%), 5 (0.4%), 3 (0.3%), 2 (0.2%), 1 (0.1%) and 1 (0.1%), respectively (Table 2). Women with multi-genotypic HPV infections were pre-excluded; the number of such women was 313, accounting for 21.2% of all of the women (i.e., 1475 cases) that were screened (Table 1).

**Table 1:** The clinicopathological characteristics of the enrolled and pre-excluded women*

**Characteristics**	**Women enrolled (n = 1162)**	**Women pre**-**excluded (n = 313)**	**p value** ^**‡**^
Age	36.1 ± 8.1	37.0 ± 7.8	0.066
Parity	2.2 ± 1.5	2.1 ± 1.2	0.118
Gravidity	0.6 ± 0.6	0.7 ± 0.7	0.346
Abnormal cervical cytological findings^†^			0.061
SCC	5 (0.4)	0 (0)	
HSIL	256 (22.0)	76 (24.3)	
LSIL	569 (49.0)	136 (43.5)	
ASC-H	123 (10.6)	25 (8.0)	
ASC-US	186 (16.9)	71 (22.7)	
AIS	0 (0)	0 (0)	
AGC-neoplastic	2 (0.2)	0 (0)	
AGC-NOS	21 (1.8)	5 (1.6)	

*The data are presented as mean ± standard deviation or number (%).

^†^SCC, squamous cell carcinoma; HSIL, high grade squamous intraepithelial lesion; LSIL, low grade squamous intraepithelial lesion; ASC-H, atypical squamous cells – cannot exclude HSIL; ASC-US, atypical squamous cells of undetermined significance; AIS, adenocarcinoma in situ; AGC-neoplastic, atypical glandular Cells, suspicious for AIS or cancer; AGC-NOS, atypical glandular cells not otherwise specified.

^‡^Two-sided Student’s t test or two-sided χ^2^ test was used as appropriate.

**Table 2:** The age-related distributional characteristics and physical statuses of the 25 HPV genotypes detected*

**HPV genotypes**	**Age groups (years)** ^**†**^										**Physical statuses** ^**‡**^	
	**20-24 (n = 66)**	**25-29 (n = 207)**	**30-34 (n = 262)**	**35-39 (n = 235)**	**40-44 (n = 212)**	**45-49 (n = 121)**	**50-54 (n = 41)**	**55-59 (n = 11)**	**60-64 (n = 5)**	**65-69 (n = 2)**	**Episomal (n = 805)**	**Integrated/Mixed** ^**§**^ **(n = 357)**
**HPV 6**	2 (3.0)	4 (1.9)	12 (4.6)	2 (0.9)	2 (0.9)	4 (3.3)	0 (0)	0 (0)	0 (0)	1 (50.0)	25 (3.1)	2 (0.6)
**HPV 11**	2 (3.0)	2 (1.0)	2 (0.8)	3 (1.3)	0 (0)	0 (0)	0 (0)	0 (0)	0 (0)	0 (0)	9 (1.1)	0 (0)
**HPV 16**	20 (30.3)	126 (60.9)	136 (51.9)	113 (48.1)	117 (55.2)	64 (52.9)	18 (43.9)	4 (36.4)	3 (60.0)	1 (50.0)	436 (54.2)	166 (46.5)
**HPV 18**	9 (13.6)	12 (5.8)	16 (6.1)	36 (15.3)	25 (11.8)	21 (17.4)	5 (12.2)	1 (9.1)	1 (9.1)	0 (0)	73 (9.1)	53 (14.8)
**HPV 26**	0 (0)	0 (0)	1 (0.4)	0 (0)	0 (0)	1 (0.8)	0 (0)	0 (0)	0 (0)	0 (0)	1 (0.1)	1 (0.3)
**HPV 31**	5 (7.6)	7 (3.4)	17 (6.5)	22 (9.4)	19 (9.0)	3 (2.5)	5 (12.2)	1 (9.1)	0 (0)	0 (0)	44 (5.5)	35 (9.8)
**HPV 33**	9 (13.6)	11 (5.3)	21 (8.0)	16 (6.8)	20 (9.4)	7 (5.8)	3 (7.3)	1 (9.1)	1 (20.0)	0 (0)	53 (6.6)	36 (10.1)
**HPV 35**	1 (1.5)	3 (1.4)	9 (3.4)	8 (3.4)	3 (1.4)	2 (1.7)	2 (4.9)	1 (9.1)	0 (0)	0 (0)	19 (2.4)	10 (2.8)
**HPV 39**	1 (1.5)	5 (1.5)	13 (2.4)	7 (5.0)	4 (3.0)	2 (1.9)	2 (1.7)	1 (4.9)	0 (0)	0 (0)	21 (2.6)	14 (3.9)
**HPV 41**	0 (0)	1 (0.5)	1 (0.4)	0 (0)	0 (0)	1 (0.8)	0 (0)	0 (0)	0 (0)	0 (0)	3 (0.4)	0 (0)
**HPV 42**	2 (3.0)	1 (0.5)	0 (0)	0 (0)	0 (0)	2 (1.7)	0 (0)	0 (0)	0 (0)	0 (0)	4 (0.5)	1 (0.3)
**HPV 45**	1 (1.5)	1 (0.5)	1 (0.4)	0 (0)	1 (0.5)	2 (1.7)	1 (2.4)	1 (9.1)	0 (0)	0 (0)	4 (0.5)	4 (1.1)
**HPV 51**	1 (1.5)	2 (1.0)	3 (1.1)	1 (0.4)	1 (0.5)	0 (0)	0 (0)	0 (0)	0 (0)	0 (0)	5 (0.6)	3 (0.8)
**HPV 52**	2 (3.0)	3 (3.0)	3 (1.4)	3 (1.1)	2 (1.3)	0 (0)	0 (0)	0 (0)	0 (0)	0 (0)	8 (1.0)	5 (1.4)
**HPV 53**	3 (4.5)	4 (1.9)	9 (3.4)	3 (1.3)	2 (0.9)	2 (1.7)	2 (4.9)	1 (9.1)	0 (0)	0 (0)	20 (2.5)	6 (1.7)
**HPV 56**	1 (1.5)	5 (0.4)	4 (1.5)	4 (1.7)	3 (1.4)	3 (2.5)	1 (2.4)	0 (0)	0 (0)	0 (0)	16 (2.0)	5 (1.4)
**HPV 58**	2 (3.0)	4 (1.9)	3 (1.1)	5 (2.1)	2 (0.9)	3 (2.5)	0 (0)	0 (0)	0 (0)	0 (0)	16 (2.0)	3 (0.8)
**HPV 59**	1 (1.5)	2 (1.0)	2 (0.8)	1 (0.4)	1 (0.5)	0 (0)	0 (0)	0 (0)	0 (0)	0 (0)	6 (0.7)	1 (0.3)
**HPV 61**	0 (0)	1 (0.5)	1 (0.4)	2 (0.9)	0 (0)	1 (0.8)	0 (0)	0 (0)	0 (0)	0 (0)	4 (0.5)	1 (0.3)
**HPV 62**	0 (0)	2 (1.0)	1 (0.4)	2 (0.9)	1 (0.5)	0 (0)	0 (0)	0 (0)	0 (0)	0 (0)	5 (0.6)	1 (0.3)
**HPV 66**	2 (3.0)	6 (2.9)	2 (0.8)	3 (1.3)	3 (1.4)	0 (0)	0 (0)	0 (0)	0 (0)	0 (0)	12 (1.5)	4 (1.1)
**HPV 68**	1 (1.5)	3 (1.4)	3 (1.1)	2 (0.9)	5 (2.4)	2 (1.7)	2 (4.9)	0 (0)	0 (0)	0 (0)	14 (1.7)	4 (1.1)
**HPV 69**	0 (0)	2 (1.0)	1 (0.4)	2 (0.9)	1 (0.5)	1 (0.8)	0 (0)	0 (0)	0 (0)	0 (0)	5 (0.6)	2 (0.6)
**HPV 74**	1 (1.5)	0 (0)	0 (0)	0 (0)	0 (0)	0 (0)	0 (0)	0 (0)	0 (0)	0 (0)	1 (0.1)	0 (0)
**HPV 93**	0 (0)	0 (0)	1 (0.4)	0 (0)	0 (0)	0 (0)	0 (0)	0 (0)	0 (0)	0 (0)	1 (0.1)	0 (0)

*The data are presented as numbers (%).

^†^No significant differences found in the age-related distribution patterns of the 25 HPV genotypes, χ2 = 217.4, p = 0.461, two-sided χ2 test.

^‡^Significant difference found between the episomal and integrated/mixed infection rates of the 25 HPV genotypes, χ2 = 47.3, p = 0.003, two-sided χ2 test.

^§^DIPS cannot differentiate the mixed HPV infection cases from the integrated infection cases.

A colposcopy-based biopsy was performed to evaluate the pathological status of the cervix. To avoid missed biopsies, patients with negative results underwent up to two additional rounds of biopsies within three months. A total of 514 precancerous/cancerous cases were thereby identified, including 286 cases of cervical intraepithelial neoplasia (CIN) I, 189 cases of CIN II, 36 cases of CIN III and 3 cases of invasive cancer. The first-round biopsy detected 477 (92.8%) cases; the second-round biopsy detected 26 (5.1%) cases; and the third-round biopsy detected 11 (2.2%) cases. The age-related distribution pattern of biopsy-diagnosed precancerous/cancerous cases is shown in Figure 2A. The number of precancerous/cancerous cases peaked at the age of 35–39 years, while the number of non-precancerous/cancerous cases peaked at the age of 30–34 years. A significant time retardation was observed between the two peak age groups (p < 0.001, two-sided χ^2^ test, Figure 2A).

**Figure 3:**
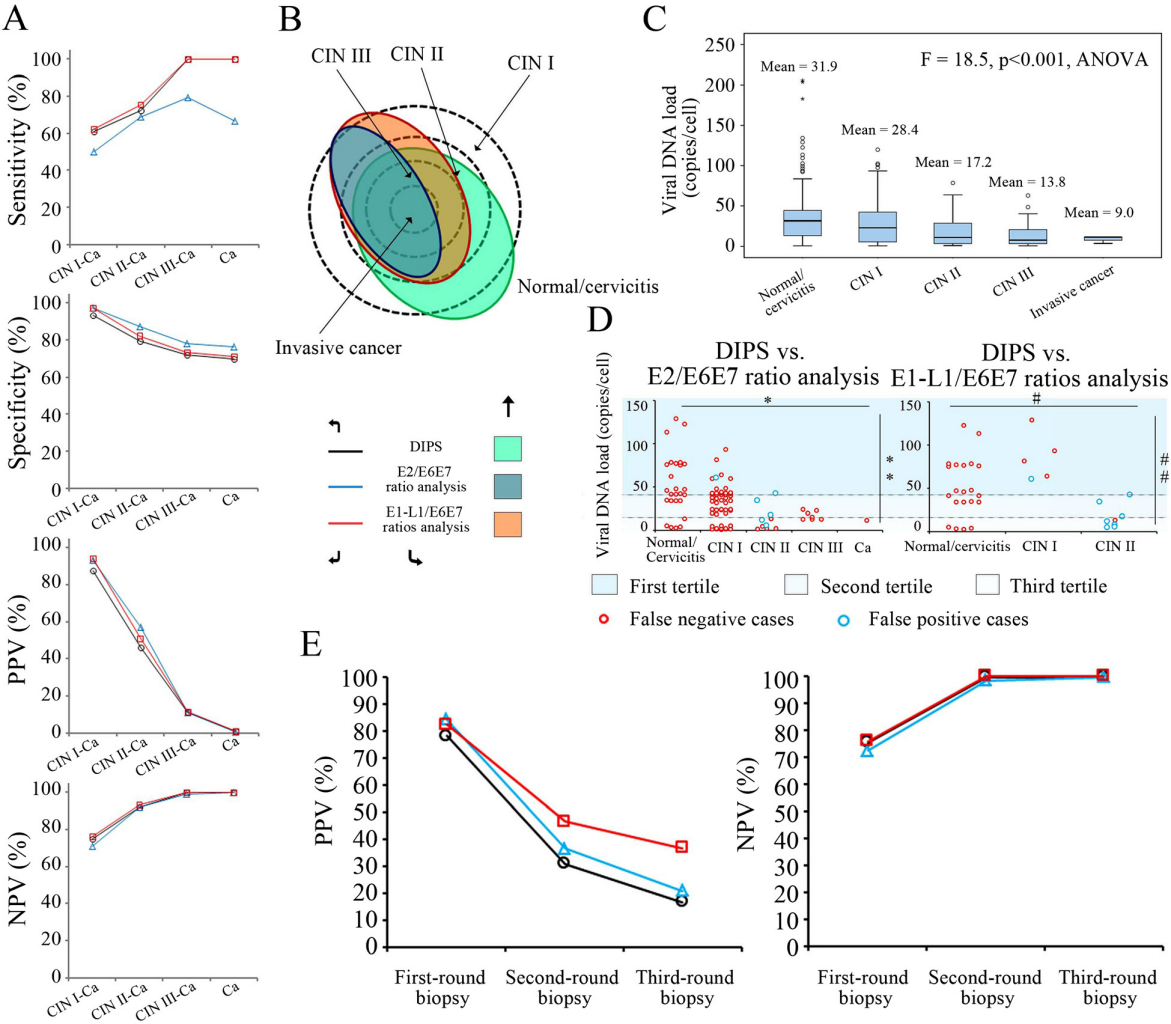
**The diagnostic performance of the copy number ratio-based techniques and DIPS. A**. Diagnostic performance of the three techniques, i.e., DIPS, the E2/E6E7 ratio analysis and the E1-L1/E6E7 ratio analysis, on the different grades of cervical precancerous/cancerous lesions. Ca, invasive cancer. **B**. Schematic representation of the detection ranges of the two copy-number-based techniques and DIPS and their relationships with the population of cervical lesions. **C**. Box plots indicating the viral load distribution characteristics among women with different levels of cervical lesions. **D**. The distribution characteristics of false-positive cases and false-negative cases that were detected by the E2/E6E7 ratio analysis and the E1-L1/E6E7 ratio analysis are shown, together with their corresponding viral loads. * and #, The differences between the numbers of false-negative cases between the five grades of cervical lesions were of statistical significance, p < 0.001, two-sided χ^2^ test. ** and ##, The differences between the numbers of false-negative cases between three viral load tertiles were of statistical significance, p < 0.001, two-sided χ^2^ test. Ca, invasive cancer. **E**. Predictive performance of the three techniques on the cervical precancerous/cancerous lesions during the three rounds of cervical biopsies.

Based on the DIPS analysis, HPV integration events (including the integrated and mixed HPV infection) were detected in 312 (312/514, 60.7%) patients with precancerous/cancerous lesions and in 45 (45/648, 6.9%) patients without such lesions. The cervical lesion-related HPV integration rates are shown in Figure 2B, and this difference was of statistical significance (p < 0.001, two-sided χ^2^ test). The age-related distribution patterns of the DIPS-confirmed (i.e., integrated/mixed infection) and DIPS-denied (i.e., episomal infection) cases are shown in Figure 2A. A similar time retardation was observed between the two peak age groups with (i.e., integrated/mixed infection) and without (i.e., episomal infection) HPV integration events (Figure 2A). Relative to the precancerous (CIN I-III)/cancerous cases that were detected by biopsies, the sensitivity, specificity, and positive (PPV) and negative (NPV) predictive values of DIPS were 60.7%, 93.1%, 87.4% and 74.9%, respectively (Figure 3A). For higher grades of lesions, that is, CIN II-III and invasive cancer, the DIPS sensitivity increased to 72.4%; when there were only CIN III and invasive cancer, the sensitivity increased to 100%; and when the lesions were restricted to invasive cancer cases, the sensitivity was 100% (Figure 3A). Nevertheless, the specificity and PPV of DIPS decreased as the CIN grades increased, especially the latter, which even decreased to 10.9% and 0.8% for lesions higher than CIN III and invasive cancer cases, respectively (Figure 3A). On the other hand, the NPVs were slightly increased as the lesion grades increased, which were 92.2% for the lesions that were equal to/higher than CIN II, 100% for the lesions that were equal to/higher than CIN III and 100% for the invasive cancer cases, displaying a similar pattern as that of the sensitivity (Figure 3A). Regarding the viral genotype-related distribution, HPV 16 and 18 gave rise to a major part of the integrated/mixed infection cases that we detected, namely, 46.5% and 14.8%, respectively (Table 2). The viral integration rates (IRs, i.e., the percentage of HPV integration cases in the women that were infected with a certain genotype of HPV), however, from the highest to the lowest, were HPV 45 (IR = 62.5%), 26 (IR = 50%), 18 (IR = 42.1%), 31 (IR = 41.8%), 33 (IR = 40.4%), 39 (IR = 40.0%), 52 (IR = 38.5%), 51 (IR = 37.5%), 35 (IR = 34.5%), 69 (IR = 28.6%), 68 (IR = 27.8%), 16 (IR = 27.7%), 53 (IR = 26.9%), 66 (IR = 25.0%), 56 (IR = 23.8%), 42 (IR = 20.0%), 61 (IR = 20.0%), 62 (IR = 16.7%), 58 (IR = 15.8%), 59 (IR = 14.3%) and 6 (IR = 3.7%) (Table 2 and Additional file 2: Figure S2). No integration events were found in the HPV 11-, 41-, 74- and 93-positive cases (Table 2). For all of the HPV genotypes, the tumorigenic rates (TRs, i.e., the percentage of CIN or invasive cancer cases in the women that were infected with a given genotype of HPV) were positively correlated with their IRs in the study population (Figure 2C).

Table 3 lists the detection results of two copy number ratio-based techniques. The viral integration events that were identified by the E1-L1/E6E7 ratio analysis were fewer by 18 cases than those identified by DIPS and were more by 63 cases than those identified by the E2/E6E7 ratio analysis (for details, see Additional file 1: Tables S2-S30). When considering DIPS as a gold-standard technique, there were 8 cases that were false-positively diagnosed by the E1-L1/E6E7 ratio analysis and 26 cases that were false-negatively diagnosed (Table 3). No cases that were identified by the E2/E6E7 ratio analysis were missed by the E1-L1/E6E7 ratio analysis (Additional file 1: Table S5). Relative to the E2/E6E7 ratio analysis, of the 63 cases that were additionally detected by the E1-L1/E6E7 ratio analysis, 96.8% (61/63) were confirmed by DIPS (Table 3). Moreover, for the 61 DIPS-confirmed cases, the viral-cellular junction sequencing results indicated that the viral disruption points were all located outside the E2 region (Additional file 1: Tables S2-S30 and in Additional file 2: Figure S1). Relative to DIPS, the multiple E1-L1/E6E7 ratio analysis exhibited a sensitivity and specificity of 92.7% and 99.0% in detecting HPV integration (integrated/mixed HPV infection) events, respectively, whereas the E2/E6E7 ratio analysis exhibited a sensitivity and specificity of 75.6% and 99.3%, respectively (Table 3). To diagnose cervical precancerous/cancerous lesions, the E1-L1/E6E7 ratio analysis exhibited a sensitivity, specificity, PPV and NPV of 60.9%, 96.0%, 92.3% and 75.6%, respectively, and the E2/E6E7 ratio analysis exhibited a sensitivity, specificity, PPV and NPV of 51.9%, 98.6%, 95.7% and 72.1%, respectively (Figure 3A). As the lesion grades increased, the diagnostic performance of the two copy number ratio analyses followed a similar pattern as that of the DIPS, except that the E2/E6E7 ratio analysis showed obvious deficiencies in its detection sensitivity for cervical lesions that were higher than CIN III and invasive cancer (Figure 3A).With respect to the detection consistencies, the κ coefficient was 0.80 between the E2/E6E7 ratio and DIPS analyses (with 93 cases of difference), 0.93 between the multiple E1-L1/E6E7 ratio and DIPS analyses (with 34 cases of difference), and 0.86 between the two copy number ratio-based techniques (with 63 cases of difference) (Figure 3B and Additional file 1: Tables S3-S5). Meanwhile, the age-, cervical lesion- and HPV genotype-related distribution patterns of the HPV integration events that were detected by copy number ratio techniques showed no significant differences from those of the DIPS analysis (Additional file 2: Figure S2).

**Table 3:** **The diagnostic efficacies of the E2/E6E7 and multiple E1-L1/E6E7 ratio analyses in relation to DIPS**

**DIPS**	E2/E6E7 ratio analysis*		E2-L1/E6E7 ratio analysis*	
	**Positive** ^**†**^	**Negative** ^**‡**^	**Positive** ^**†**^	**Negative** ^**‡**^
**All women (1162 cases)**				
**positive** (n = 357)	270 (75.6)	87 (24.4)	331 (92.7)	26 (7.3)
**negative** (n = 805)	6 (0.7)	799 (99.3)	8 (1.0)	797 (99.0)
**Women in the highest tertile (387 cases, viral DNA load ≥ 36.7 copies/cell)** ^**§**^				
**positive** (n = 69)	31 (44.9)	38 (55.1)	52 (75.4)	17 (24.6)
**negative** (n = 318)	2 (0.6)	316 (99.4)	2 (0.6)	316 (99.4)
**Women in the middle tertile (387 cases, 12.7 copies/cell ≤ viral DNA load < 36.7 copies/cell)** ^**§**^				
**Positive** (n = 123)	97 (78.9)	26 (21.1)	118 (95.9)	5 (4.1)
**negative** (n = 264)	2 (0.8)	262 (99.2)	2 (0.8)	262 (99.2)
**Women in the lowest tertile (388 cases, viral DNA load <12.7 copies/cell)** ^**§**^				
**positive** (n = 165)	142 (86.1)	23 (13.9)	161 (97.6)	4 (2.4)
**negative** (n = 223)	2 (0.9)	221 (99.1)	4 (1.8)	219 (98.2)

*The data are presented as numbers (%).

^†^The positive cases include both the mixed and integrated HPV infection cases that were detected by the copy number ratio-based techniques.

^‡^The negative cases indicate the episomal HPV infection cases that were detected by the copy number ratio-based techniques.

^§^The women were divided into tertiles according to their viral DNA loads as determined by real-time PCR in the cervical samples.

To confirm whether the viral load affects the detection consistency between the copy number ratio-based techniques and DIPS, we divided HPV-infected cases into tertiles based on the E6E7/ACTB ratios. In the highest (first) tertile, relative to the results of DIPS, both the numbers and percentages of false-negative cases in the two copy number ratio-based analyses peaked (38 and 55.1% for the E2/E6E7 ratio analysis and 17 and 24.6% for the E1-L1/E6E7 ratio analysis) (Table 3 and Figure 3D). Additionally, there were 2 (0.6%) and 2 (0.6%) false-positive cases raised by the copy number ratio-based analyses in this tertile, respectively (Table 3 and Figure 3D). In the middle (second) and lowest (third) tertiles, the numbers and percentages of false-negative cases decreased in both of the copy number ratio-based analyses (Table 3 and Figure 3D); however, false-positive cases still existed, and even in the E1-L1/E6E7 ratio analysis the number and percentage of false-positive cases increased by approximately 2-fold compared to those that were observed in the first tertile (Table 3). Most of the false-negative cases that were caused by the copy number ratio-based techniques occurred in patients with no precancerous/cancerous changes or low-grade CIN (CIN I), but the false-positive cases were more frequently observed in patients with higher grades of CINs (CIN II and III) and invasive cancer (Figure 3D). Correspondingly, the number of CIN and cervical cancer cases showed tended to occur at lower viral load tertiles, but normal cervix/cervicitis cases more often appeared in the first and second tertiles (p < 0.001, ANOVA, Additional file 2: Figure S3). The observed viral load difference between the cervical lesion groups (normal/cervicitis, CIN I-III, invasive cancer) was of statistical significance (p < 0.001, ANOVA, Figure 3C).

Because our purpose for detecting integrated HPV infection in the cervix was to promote the early diagnosis (i.e., as a triage test) of cervical precancerous/cancerous lesions, we compared the efficacies of the three techniques, namely, DIPS, E2/E6E7 ratio analysis and multiple E1-L1/E6E7 ratio analysis, in predicting CIN or invasive cancer cases among the enrolled women. As shown in Figure 3E, the PPVs of the three techniques for CINs were 78.2% (DIPS), 84.1% (E2/E6E7) and 82.3% (E1-L1/E6E7) during the first-round biopsy, respectively. The NPVs were similar, namely, 75.4% (DIPS), 72.3% (E2/E6E7) and 75.9% (E1-L1/E6E7), respectively. During the second-round biopsy, the PPVs of the three techniques decreased to 30.8% (DIPS), 36.4% (E2/E6E7) and 45.5% (E1-L1/E6E7), respectively, while their NPVs all increased, namely, 99.7% (DIPS), 99.1% (E2/E6E7) and 100% (E1-L1/E6E7), respectively. For the third-round biopsy, the PPV of DIPS decreased to 16.7%, while the PPVs of the two copy number ratio-based analyses were 20.8% (E2/E6E7) and 36.7% (E1-L1/E6E7), respectively. However, the NPVs of the three techniques were maintained stable at 99.7% (DIPS), 99.1% (E2/E6E7) and 100% (E1-L1/E6E7), respectively. Although DIPS could detect more cases of integrated HPV infection than could the copy number ratio-based analyses, our results indicate that this performance was not necessarily associated with the ability to predict cervical precancerous/cancerous lesions. Indeed, the E2/E6E7 ratio analysis and E1-L1/E6E7 ratio analysis, which exhibited moderate and lower sensitivities for HPV integration events, respectively, both achieved higher PPVs and/or NPVs throughout the three rounds of biopsies. The E1-L1/E6E7 ratio analysis especially exhibited the supreme PPVs and NPVs before the second- and third-round biopsies, indicating its excellent value in directing repeated biopsies in the suspected women.

We examined the detection reproducibility of the three techniques one month after a first-round test. Following two additional rounds of analyses on the remaining cervical samples preserved at −20°C, the coefficients of variation (CVs) of each copy number ratio analysis were obtained, which were 5.1%-7.8% (E1/E6E7), 3.9%-5.6% (E2/E6E7, for both the E2/E6E7 ratio- and E1-L1 ratio-based techniques), 2.3%-6.9% (E4E5/E6E7), 2.9%-8.2% (L2/E6E7) and 3.1%-7.1% (L1/E6E7), respectively. Using CV = 10% as a threshold, the quantification stability of the two techniques was acceptable. Moreover, the detection consistency (i.e., reproducibility) of the three rounds of tests was 100% for both of the techniques. However, 79 (79/357, 22.1%) of the integrated/mixed infection cases that were identified during the first-round DIPS failed to be detected again during the second- or third-round DIPS. Meanwhile, two of the so-called false-positive cases (Patient Nos. 179275 and 624287, Additional file 1: Tables S8 and S25) as detected by the copy number ratio-based techniques were proven to be true positives by the second-round DIPS (but it failed again in the third-round detection). In total, three cases were thus corrected by a second- or third-round DIPS (Table 4 and Additional file 1: Tables S8, S12 and S25). Therefore, the detection reproducibility of this technique was 92.9% (1080/1162). In addition, we re-examined the remaining five cases that were denied by DIPS but positively diagnosed by E2/E6E7 ratio analysis or E1-L1/E6E7 ratio analysis using a modified version of DIPS in which the endonucleases TaqI and Sau3AI were substituted with Csp6I. As a result, another case (Patient No. 189543, Additional file 1: Table S9) was confirmed as a true positive. The DNA sequencing results indicate there were no TaqI or Sau3AI recognition sites within the 2–3 kb region franking the viral-cellular junction of this case (Additional file 2: Figure S1).

**Table 4:** The reproducibility of DIPS for triplicate testing of cytological samples after a one-month preservation*

**DIPS rounds**	**First-round DIPS (+) (n = 357)**	**First-round DIPS (−) (n = 805)**		
		**E2/E6E7 ratio analysis (+) & E1-L1/E6E7 ratio analysis (+) (n = 6)**	**E2/E6E7 ratio analysis (−) & E1-L1/E6E7 ratio analysis (+) (n = 2)**	**E2/E6E7 ratio analysis (−) & E1-L1/E6E7 ratio analysis (−) (n = 797)**
**Second-round**	Confirmed: 299	Confirmed: 5	Confirmed: 2	Confirmed: 797
	Denied: 58	Denied: 1	Denied: 0	Denied: 0
	Reproducibility between 1st- and 2nd-round DIPS: 94.9%			
**Third-round**	Confirmed: 281	Confirmed: 5	Confirmed: 1	Confirmed: 797
	Denied: 76	Denied: 1	Denied: 1	Denied: 0
	Reproducibility between 1st- and 3rd-round DIPS: 93.3%			
**Second- and Third-round**	Consistently confirmed: 278	Consistently confirmed: 4	Consistently confirmed: 1	Consistently confirmed: 797
	General reproducibility: 92.9%			

*The reproducibility was calculated by dividing the number of cases that were confirmed by two or three rounds of DIPS by the total number (n = 1162) of tested cases.

## Discussion

Herein, we offered the first insight into a novel, practicable and multigenotype-oriented tool for detecting HPV integration that was developed based on an existing, single-genotypic, research-based technique, i.e., the E2/E6E7 ratio analysis. In the population of enrolled women, this novel technique has not only manifested a similar performance to that of DIPS, a recognized gold-standard detection technique (Figure 3A), but also featured a simplified process of operation, producing a workload that is about one fourth that of DIPS (note: we assumed that the workload is proportional to the number of PCR reactions required to perform a HPV integration test, while the workload generated by DIPS via sample pretreatment and PCR product sequencing has not been taken into account) (Figure 1B).

In this study, the E1-L1/E6E7 ratio-based technique was used to evaluate a total of 25 HPV genotypes (compared to the E2/E6E7 ratio analysis and DIPS) for their physical statuses. The number of HPV genotypes that were evaluated, to the best of our knowledge, has reached the maximum among known studies concerning integrated HPV infection. In previous studies, only two to five genotypes of HPVs have been evaluated via three major techniques, namely, APOT, DIPS and the E2/E6E7 ratio analysis. However, such a narrow genotype range does not satisfy the clinical demands, which could be associated with a high misdiagnosis rate in women that are infected with other HPV genotypes. In a nine-country collaborative international study, Muñoz et al. detected 30 genotypes among the 1998 cases of cervical HPV-infected women (including 1739 cervical cancer and 259 simple infection cases) [17]. Similarly, in this study, we detected 25 genotypes in the enrolled 1162 HPV-positive women, 19 of which were in accordance with those that were reported by Muñoz et al., while the remaining six (HPV 41,61, 62, 69, 74 and 93) were peculiar to the enrolled population, reflecting a characteristic genotypic spectrum in Chinese women [17]. Together with the findings of Muñoz et al., our results suggest that only the techniques that are applicable to 20 to 30 HPV genotypes can meet the practical need. We, therefore, built the primer and probe library (Additional file 1: Table S1) to implement this aim, which can be extended to additional HPV genotypes under necessary conditions. This library comprises a common base in which to compare the two copy number ratio-based techniques as well as the standard technique DIPS in multigenotypic HPV.

It is noteworthy that we only adopted the primer and probe library but not the traditional degenerate (i.e., consensus) primer strategy for adapting the three techniques, i.e., DIPS, the E2/E6E7 ratio analysis and the E1-L1/E6E7 ratio analysis, to detect multigenotypic HPV [18-21]. Moreover, we used the sequence-specific TaqMan probe but not DNA dyes, such as SYBR Green, BEBO, SYTO, etc. [22], to determine the gene copy number. Although degenerate primers have been widely accepted by previous HPV detection tools, and the addition of DNA dyes is a popular choice for lowering the expense of a real-time PCR test, the quantification bias could be inevitably induced by the mis-binding of these degenerate primers to unexpected cellular/viral genomic sequences and the insertion of DNA dyes to non-specific primer dimers and/or mistakenly amplified products [18-22]. Therefore, the adoption of sequence-specific primers and probes could solve these problems. In addition, Depuydt et al. reported that the L1 degenerate primer pair MY09/11 varies in its ability to bind to L1 sequences of different HPV genotypes, leading to a reduced sensitivity for several HPV genotypes [23]. This observation further demonstrates the necessity of adopting genotype-specific primers and probes in the multigenotypic detection system. Meanwhile, to avoid systemic bias between techniques, the primers and probes that were designed to recognize a given viral sequence target were identical in all three techniques (Figure 1A and Additional file 1: Table S1). These measures allow the results that were obtained from each technique to be compared in a homogenous technological background.

Based on the three compared techniques, i.e., the DIPS analysis and the two copy number ratio-based analyses, the importance of applying HPV integration tests as tools to prevent cervical cancer was consistently demonstrated by a panel of mutually corroborative lesion-, genotype- and age-related distributional characteristics of integrated/mixed HPV infections in the enrolled women. Our results indicate that, with increasing lesion grades, the percentages of integrated/mixed infection cases increased gradually (Figure 2B). This finding agrees with previous epidemiological studies, which revealed similar patterns of integration events in HPV 16-, 18-, 31-, 33-, 45-, 52- or 58-infected women [7,24-26]. Nevertheless, our results have generalized this ad hoc rule from one that was only valid for a few HPV genotypes to a more common form that is suitable for 25 or more HPV genotypes (Figure 2B and Additional file 2: Figure S2). Meanwhile, based on the same database, we are now able to unveil another epidemiological rule, that is, the integration ability of a given HPV genotype is positively correlated with its tumorigenic potential (Figure 2C). This assertion can be seen as a direct inference of the prior rule of the relationship between the integration rate and the cervical lesion grade, which is specific to the oncological behavior of each HPV genotype. Frequent integration events can result in the early occurrence of cervical cancer. In a study conducted by Vinokurova et al., the integration rates of HPV 16, 18, 31, 33, and 45 were inversely correlated with the ages of onset of cervical cancer [26]. Unfortunately, due to the limited number of cancer cases (i.e., 3 cases) in this study, we cannot confirm this finding in our population, although a larger number of viral genotypes were involved. However, we did observe an overlap of the peak age groups of women with precancerous/cancerous lesions and those with integrated/mixed HPV infections, which has been confirmed by DIPS, the E2/E6E7 ratio analysis and the E1-L1/E6E7 ratio analysis (Figure 2A). Additionally, a time interval was observed between the peak age groups of women with precancerous/cancerous lesions and those of unaffected women and another similar inter was found between the peak age groups of episomal- and integrated/mixed-infection cases, indicating that HPV integration and cervical carcinogenesis develop in a synchronous manner (Figure 2A). These lines of evidence further demonstrate the necessity of adopting an HPV integration test as a triage method to identify high-risk women and provide them timely treatment and/or intensive follow-up. These women were fundamental for our comparison of the two copy number ratio-based techniques.

Multigenotypic DIPS - a common reference platform was established in our study for comparing the respective diagnostic accuracies of the E2/E6E7 ratio analysis and the E1-L1/E6E7 ratio analysis in the enrolled women. The original version of DIPS was designed for two genotypes of HPVs, namely, HPV 16 and 18 [12]. The operating principle of DIPS is to sequence the viral-cellular junction in the integrated/mixed HPV-infected cells and, therefore, to provide objective evidence for viral integration events [12]. Theoretically, this method can exhaustively determine any possible insertion points of viral DNA in the cellular genome. Therefore, in many previous studies, DIPS has been treated as a gold-standard technique, especially for evaluating the diagnostic correctness of the E2/E6E7 ratio analysis [5,7,14,15,24,25,27,28]. In this study, DIPS unsurprisingly detected 18 and 81 additional cases compared to those that were identified by the E2/E6E7 ratio analysis and the E1-L1/E6E7 ratio analysis, accounting for 5% (18/357) and 22.7% (81/357) of the total integrated/mixed infection cases, respectively (Table 3). Considering the κ value as a measurement for evaluating technological similarity, we observed that the technological characteristic of the E1-L1/E6E7 ratio analysis is more similar to that of DIPS but not to that of its prototype technique the E2/E6E7 ratio analysis (Additional file 1: Tables S3-S5). This result can be considered a major advantage of the copy number ratio-based techniques following the extension of the originally limited detection region to a whole viral genome in detecting HPV integrated/mixed infection. Previously, using the whole-genome sequencing technique, Wang et al. found that there could be 67-81% integration events occurring outside the E2 gene region [29]. Our study supports their findings and demonstrates that the non-E2 integration events represent a frequently encountered phenomenon in the clinic, which could be at least 17.1% (61/357) of the total integrated/mixed infection cases. These integration events were also associated with a rate of precancerous/cancerous lesions and, therefore, gave rise to a non-negligible group of high-risk women (Figure 3D, Additional file 1: Tables S6-S30). As such, the E1-L1/E6E7 ratio analysis is superior to the E2/E6E7 ratio analysis as a triage test to screen for non-E2 HPV integration cases.

In the detection of integrated/mixed HPV infection, the two copy number ratio-based techniques were being confronted with the same problem: the complete viral genome of episomal HPV copies can cover up a few viral sequence deletions that are caused by the integration events, leading to an indifferent copy number ratio to that of episomal infection and, thereafter, to false-negative cases [30,31]. In this study, we used DIPS to assess the influence of episomal viral copies on the diagnostic correctness of the two copy number ratio-based techniques. As a result, of the 357 integrated/mixed infection cases that were identified by DIPS, 26 (7.3%) were not detected by either of the two compared techniques (Table 3), indicating a systemic error caused by the covering-up phenomenon in a background of excessive episomal DNA. To confirm this hypothesis, we analyzed the relationship between the viral DNA load and the false-negative rate of the copy number ratio-based analyses (Table 3, Figure 3D). The obtained results indicate a positive correlation between the amount of episomal viral DNA and the number of false-negative cases that were detected by the E2/E6E7 ratio analysis or the E1-L1/E6E7 ratio analysis, whereby the interference effect of the episomal copies could be ascertained (note: most of the cases in the highest tertile were denied by DIPS; therefore, they were at an episomal infection status) (Table 3, Figure 3D). Given this result, it seems that the copy number ratio-based techniques, regardless of form, cannot bypass an inherent technical deficiency of this type. However, if considered in an opposite way, our result may be interpreted as the copy number ratio-based techniques should be preferentially used to detect HPV integration events among women with lower viral DNA loads, thereby reducing its false-negative rate. In fact, previous studies have demonstrated that the viral integration process is often accompanied by the lowering of the viral DNA load as well as the increasing of the cervical lesion grade [7,24-26,32-34]. The reason for this phenomenon could be the methylation-mediated silence or insertion-caused disruption of the viral genome replication-related genes, such as E1 and E2, or the activation of viral oncogenes E6 and E7 after the integration event [35,36]. Here, our study supportively confirms that lower viral load tertiles are associated with more integration events and higher incidence rates of precancerous/cancerous lesions (Figure 3C and Additional file 2: Figure S3). Therefore, the cases that were identified by the copy number ratio-based techniques were generally more severe in their pathological conditions compared to those that were detected by DIPS because most of these cases had lowered viral loads and were near to the canceration stage (also see the analyses in the following paragraphs) (Figure 3D). In addition, our study demonstrates that the peak age group of integrated/mixed HPV infection emerged approximately five years after the peak age group of episomal HPV infection (Figure 2A). Therefore, it is reasonable to use the long-term (e.g., >six months) HPV-infected women as the target population of the copy number ratio-based methods. This measure can be helpful to reduce the interference effect of episomal copies and improve the detection accuracy of the two copy number ratio-based techniques.

The enrolled population of this study consisted of women with HPV infection > six months who had positive findings in their cervical cytological examinations. Previous studies indicated that 30%-60% of these women would be eventually diagnosed as normal or cervicitis cases [37,38]. Therefore, colposcopy and cervical biopsy are not so necessary, at least for some of the women, and can induce unnecessary stress. In this study, we first tested the triage role of the gold-standard technique, DIPS. For high-grade lesions and invasive cancer, DIPS displayed satisfactory sensitivity: 66.7% (CIN II), 100% (CIN III) and 100% (invasive cancer) (Figure 2B); the DIPS specificity was 93.1% for all of the cases with no precancerous/cancerous lesions. However, for the CIN I cases, the DIPS sensitivity was only 51.4%; moreover, DIPS detected 45 (6.9%) integrated/mixed infection cases in normal/cervicitis cases (Figure 2B). The latter two phenomena comprise a major reason for erroneous diagnosis/prediction by DIPS in triaging cervical lesion cases (Figure 3A). Regarding the performances of the two copy number ratio-based analyses, the E1-L1/E6E7 ratio analysis exhibited sensitivity identical to that of DIPS (60.9% vs. 60.7%) for all grades of precancerous/cancerous lesion cases; the sensitivities of both the E1-L1/E6E7 ratio analysis and DIPS were superior to that (51.9%) of the E2/E6E7 ratio analysis. Nevertheless, regarding specificity, PPV and NPV, the two copy number ratio-based techniques exhibited similar performances to those of DIPS (Figure 3A). Inevitably, no matter which technique is performed, a proportion of lower grade (CIN I and II) lesion cases or non-precancerous/cancerous lesion cases will be misdiagnosed. Previous studies indicated that not all CIN cases occur due to HPV integration; conversely, not all integration events result in precancerous/cancerous lesions [7-9,24-28]. Moreover, integrated HPV requires a period of time to exert its oncological function and induce canceration of the affected cells [2,3,11]. Therefore, it is reasonable to conclude that no HPV integration detection technique can achieve 100% sensitivity or specificity in precancerous/cancerous cervical lesions, especially for CIN I and II cases. However, because it is more difficult to eradicate integrated HPV DNA from the cellular genome, CINs with integrated/mixed infections could be disposed to persist or progress their conditions, while CINs with episomal HPV infections can regress spontaneously [39,40]. Therefore, more attention should be paid to women with integrated/mixed HPV infections, regardless of the grade of their cervical lesions. To explain the difference in sensitivity between the two copy number ratio-based techniques, we refer to the detection bias between restrictive integration tests and systemic integration tests. A restrictive integration test (i.e., that detects DNA fragments of a partial viral genome), such as APOT (a technique that detects the viral-cellular junction within the transcripts of the E6E7 oncogene) and the E2/E6E7 ratio analysis, cannot effectively examine all existing integrated/mixed infections [6,12-15,26]. For example, Vinokurova et al. detected the integration events in only 14%-92% of cervical cancer patients using APOT [26], and Arias-Pulido et al. gained an integration rate of 57.7%-65% in HPV 16-infected women suffering from cervical cancer using an E2/E6E7 ratio-based technique [14]. In contrast, using DIPS, a systemic integration test (i.e., detecting DNA fragments of the whole viral genome), Luft et al. demonstrated that the integration rate of HPV was close to or approached 100% in HPV 16- and 18-infected patients with cervical cancer [12]. These findings indicate that only a systemic integration test could reach 100% sensitivity in predicting cervical cancer cases. Our study, therefore, confirmed another systemic integration test, the E1-L1/E6E7 ratio analysis, as capable of sensitively identifying the women with high-risk cervical precancerous/cancerous lesions based on a wider range of HPV genotypes and that is more eligible than the E2/E6E7 ratio analysis.

Cervical biopsy is a key method to acquire direct evidence of precancerous/cancerous lesions. This process provides reliable guidance to develop a proper treatment scenario. However, we and other groups have demonstrated a rate of missed biopsy, especially in women with no visible lesions in the cervix [41-44]. In this study, this rate reached 5.1% during the first-round biopsy and 2.2% during the second-round biopsy. Because the three biopsies were very close in time to one another (within three months), the missed cases should not be considered newly developed during the examination interval. A rational reason for this phenomenon is the “jumping”-style distributional characteristic of the colony-forming cervical lesions, which compel the “multipoint” biopsy to function in a randomized manner to capture disease sites. Unlike the cervical biopsy, the HPV integration test adopts as its experimental material exfoliated cells, which can be easily obtained by brushing the outer orifice of the cervix. The obtained exfoliated cells are from all of the layers of epithelium located in the entire cervical region. Therefore, this test is more efficient than the multipoint tissue-punching process of cervical biopsy for acquiring diseased cells, which makes the HPV integration test a comprehensive method for assessing cervical lesions. In our study, after a first round of biopsy, the remaining undetected cases only accounted for a very small proportion of the women, i.e., 7.3% (5.1% + 2.2%). We demonstrated that, using the HPV integration test, most of these women could be identified before a second-round biopsy, while only a few would be missed. The NPVs of the three techniques were all greater than 99%, and the PPVs varied between 30% and 45% (Figure 3D). These PPVs indicate that the efficacy of a second-round biopsy could be increased by approximately four- to six-fold (compared to the CIN detection rate during the second-round biopsy) with the assistance of integration tests. Moreover, given the fact that, even in the third-round biopsy, the HPV integration test exhibited a 16% to 36% PPV, and >1% women were afterwards diagnosed with precancerous/cancerous lesions, our results suggest that the HPV-positive women with cervical cytological abnormalities, such as HSIL, LSIL, ASC-H and ASC-US, deserve repeated biopsies to rule out potential life-threatening lesions [44]. The delay or neglect of this task could lead to disease progression and poorer prognosis. However, a ridiculous phenomenon was observed in our study; that is, the most sensitive technique, DIPS, showed much lower PPVs as a triage test compared to the two copy number ratio-based techniques (Figure 3E). To provide a reasonable explanation, we noted that some integrated/mixed HPV infection cases, although having no identifiable precancerous/cancerous lesions, were detected by DIPS but were otherwise denied by the copy number ratio-based techniques. The majority of these cases were located at the highest tertile of the viral load; therefore, these cases were difficult to detect using only a copy number ratio-based method (Figure 3D). These cases should occur during the early stage of integration, as no significant changes were found in their affected tissues. They comprised the major source to undermine the PPV performance of DIPS in cervical lesion sufferers. In addition, similar PPV phenomena were also observed between the E1-L1/E6E7 ratio analysis and the E2/E6E7 ratio analysis. The former displayed PPVs that were 9.1% and 15.9% higher than those of the latter during the second- and third-round biopsies, respectively. However, the number of invalid cases that were produced by the over-sensitive detection of non-precancerous and -cancerous lesions in the E1-L1/E6E7 ratio analysis was six greater than was that produced by the E2/E6E7 ratio analysis during the second-round biopsy and became equal during the third-round biopsy (Figure 3D and E and Additional file 2: Figure S3). Therefore, the improvement of PPVs by the E1-L1/E6E7 ratio analysis should be explained by its increased efficacy in detecting CIN I-III and/or cervical cancer cases per se but not the reduced valid cases (refer to Figure 3D). In thus way, the E1-L1/E6E7 ratio analysis achieved the highest predictive ability, making it a useful triage test to direct repeated biopsies in the suspected women.

The clinically collected samples usually need to be preserved for a period of time until they can be examined or re-examined under necessary conditions. A clinically applicable test, therefore, is expected to be able to maintain its detection ability as the sample quality begins to decrease. We re-examined the DNA extractions of cervical samples after one-month of preservation at −20°C. A lowered technical reproducibility was observed only among the results of triplicate DIPS (Table 4), while both of the two copy number ratio-based analyses maintained 100% detection consistency for three technical repeats. We postulated that the reason should reside in the different tolerances of these three techniques to DNA degradation. The DIPS technique requires high-quality DNA templates to maintain its detection ability, where no nucleotide nicks or breaks should exist in the sequences flanking or spanning the viral-cellular junctions. Once DNA degradation occurs, the remaining viral-cellular junction-containing sequences must be destroyed so that they cannot be amplified by DIPS as effectively as in freshly isolated DNA, leading to a loss of detection accuracy in the long-term preserved samples. In contrast, the copy number ratio analysis can persistently maintain its technical stability under DNA degradation conditions because the ratios of each pair of target segments are unaffected in a homogenously decayed genome DNA background. Regarding the individual ratios that comprise the entire copy number ratio analysis, their CVs were all lower than 10%, which agree with the acknowledged international standard on the quantitative stability of techniques that are used for clinical detection [45-47]. A few cases were missed by the first-round DIPS and were detected by the second- or third-round DIPS. This phenomenon reflected the random nature of the DIPS detection. The efficacy of DIPS depends on the successful ligation between the adaptors and the ends of the viral-cellular junction-containing sequences [12]. Sometimes, due to uncertain reasons (e.g., enzyme inactivation, too short reaction time and inadequate sample DNA), the ligation process could fail and lead to missed detection. These technical or artificial faults were corrected when we repeated the DIPS procedure; therefore, some incorrect results of the first-round DIPS can be identified and redressed. In addition, a change in the restriction endonucleases in DIPS also resulted in the identification of new cases, reflecting a sequence distance-limited detection ability of this technique. As we changed the restriction enzyme, the linear distance between the adapter and primer-binding sequence site was shortened to a general PCR reachable range [48,49]; therefore, the viral-cellular junction was amplified and sequenced. On this aspect, the two copy number ratio-based techniques hold a natural and predominant advantage.

Our study of women with single-genotypic HPV infections has its limitations. Previous studies indicated that women with multi-genotypic HPV infections can account for 10% to 20% of the total cervical HPV-positive cases [17,27]. This population is worthy of specific attention, as the detection objectives are beyond the ability of presently available HPV integration tests. Future studies should focus on developing a PCR array (viral genotypes × ratio targets) to detect the integration statuses of various HPV in a multi-genotypically infected woman. Although the experimental workload could thus be doubled, a more complete detection coverage on the goal population can be achieved using this new advanced PCR array-based copy number ratio-based analysis, whereby a cooperative effect of different HPV genotypes on the carcinogenesis of cervical epithelium can be explored and understood. Moreover, the multiple E1-L1/E6E7 ratio analysis has adopted four more PCR reactions than those of a traditional E2/E6E7 ratio analysis, whereas the cost-effectiveness of this modification is not yet known and should be estimated in future studies. Nevertheless, compared to DIPS, the cost of the E1-L1/E6E7 ratio analysis is significantly decreased (Figure 1B). In light of the similar diagnostic performance as well as the high detection consistency between the DIPS and E1-L1/E6E7 ratio analyses (Figures 3A, Additional file 2: Figure S2, Additional file 1: Tables S2-S5), the advantage of using the E1-L1/E6E7 ratio analysis to serve as a triage test is convincible, indicating its popularization in clinical practice.

## Conclusions

In conclusion, our study demonstrates that the multiple E1-L1/E6E7 ratio analysis is more sensitive and predictive than its prototype the E2/E6E7 ratio analysis, and it is also a more convenient and stable technique if the gold-standard technique, DIPS, is used as a reference. This technique is capable of meeting the growing demands for detecting integrated/mixed HPV infection cases in the current clinic. Meanwhile, regarding identifying the high-risk women of cervical cancer and selecting candidates for colposcopy and cervical biopsy, this technique possesses a substantial value as a triage test, which can effectively reduce the range of the target population, thereby concentrating limited medical resources to those in the most need and ameliorating unnecessary stress in low-risk women. At last, as a novel, extensible and broad-spectrum technique, the multiple E1-L1/E6E7 ratio analysis could be used by more HPV integration-related studies to explore the epidemiological mechanisms of cervical cancer that is induced by a score of HPV genotypes.

## Additional files

Additional file 1:
**Supplementary Tables. Table S1** lists the primers, probes and restriction enzymes that were used in the multigenotypic DIPS, the E2/E6E7 ratio analysis and the E1-L1/E6E7 ratio analysis in this study. **Table S2** describes the detection results of the three techniques and compares their consistencies. **Tables S3-S5** provides a detailed comparison of the detection results between each pair of the three techniques. **Tables S6-S30** indicates the detection results of the three techniques in each individual woman. Additional file 2:
**Supplementary Figures. Figure S1** schematically depicts the principal of DIPS and provides some sequencing-verified samples of the E2-related and non-E2 HPV integration events. In addition, an example of the undetected cases of DIPS, which was corrected by changing a restriction enzyme, is displayed. **Figure S2** shows the age-, genotype- and cervical lesion-related distribution patterns of the integrated/mixed HPV infection that was detected by the three techniques among the enrolled women. **Figure S3** describes the distribution characteristics of the DIPS-, E2/E6E7 ratio analysis- and E1-L1/E6E7 ratio analysis-detected cases among the viral load-cervical lesion two-dimensional space, where the y-axis consists of three DNA load tertiles, and the x-axis consists of five biopsy-identified lesion grades.

## Abbreviations

AGC-neoplastic: Atypical glandular cells, suspicious for AIS or cancer
AGC-NOS: Atypical glandular cells not otherwise specified
AIS: Adenocarcinoma in situ
APOT: Amplification of papilloma virus oncogene transcripts
ASC-H: Atypical squamous cells – cannot exclude HSIL
ASC-US: Atypical squamous cells of undetermined significance
CIN: Cervical intraepithelial neoplasia
DIPS: Detection of integrated papillomavirus sequences
HPV: Human papillomavirus
HSIL: High grade squamous intraepithelial lesion
IR: Integration rate
LCR: Long control region
LSIL: Low grade squamous intraepithelial lesion
PCR: Polymerase chain reaction
TR: Tumorigenic rate
SCC: Squamous cell carcinoma
