# Supplementary material for: Diagnosis of 25 genotypes of human papillomaviruses for their physical statuses in cervical precancerous/cancerous lesions: a comparison of E2/E6E7 ratio-based vs. multiple E1-L1/E6E7 ratio-based detection techniques
Source: J Transl Med. 2014 Oct 2;12:282. doi: 10.1186/s12967-014-0282-2 (PMC4192431; doi:10.1186/s12967-014-0282-2)
Supplement: Additional file 1: — Supplementary Tables. Table S1 lists the primers, probes and restriction enzymes that were used in the multigenotypic DIPS, the E2/E6E7 ratio analysis and the E1-L1/E6E7 ratio analysis in this study. Table S2 describes the detection results of the three techniques and compares their consistencies. Tables S3-S5 provides a detailed comparison of the detection results between each pair of the three techniques. Tables S6-S30 indicates the detection results of the three techniques in each individual woman. [file 12967_2014_282_MOESM1_ESM.pdf]

Supplementary Tables

Table S1. Primers and probes that were used in the DIPS, the E2/E6E7 ratio analysis and the multiple E1-L1/E6E7 ratio analysis for the 25 HPV genotypes.*

HPV Genotypes		E1		E2		E4E5		L2		L1		E6E7	
		**5' at**	**3' at**	**5' at**	**3' at**	**5' at**	**3' at**	**5' at**	**3' at**	**5' at**	**3' at**	**5' at**	**3' at**
**HPV 6**	Primer-F	1504	1528	2752	2773	3278	3301	4947	4792	5841	5863	119	142
	Primer-R	2470	2496	3665	3684	4427	4447	5849	5871	6675	6698	936	959
	Probe	1779	1803	2964	2990	3672	3693	5124	5151	6083	6108	160	183
**HPV 11**	Primer-F	1503	1527	2751	2772	3277	3300	4937	4962	5822	5844	118	141
	Primer-R	2469	2495	3661	3680	4420	4440	5830	5852	6659	6682	935	958
	Probe	1778	1802	2963	2989	3668	3689	5114	5141	6064	6089	159	182
**HPV 16**	Primer-F	1532	1556	2783	2804	3309	3332	4768	4793	5692	5714	117	140
	Primer-R	2498	2524	3696	3715	4239	4259	5700	5722	6535	6558	971	994
	Probe	1807	1831	2995	3021	3703	3724	4945	4972	5940	5965	158	181
**HPV 18**	Primer-F	1603	1627	2857	2878	3386	3409	4773	4798	5667	5689	124	147
	Primer-R	2569	2595	3761	3780	4247	4267	5675	5697	6510	6533	1017	1040
	Probe	1878	1902	3069	3095	3768	3689	4950	4977	5915	5940	165	188
**HPV 26**	Primer-F	1516	1540	2764	2785	3290	3313	4718	4743	5642	5664	110	133
	Primer-R	2482	2508	3710	3729	4192	4212	5650	5672	6485	6508	978	1001
	Probe	1791	1815	2976	3002	3717	3738	4898	4925	5887	5912	151	174
**HPV 31**	Primer-F	1470	1494	2721	2742	3247	3270	4688	4713	5606	5628	121	144
	Primer-R	2436	2462	3655	3674	4174	4194	5614	5636	6452	6475	965	988
	Probe	1745	1796	2933	2959	3662	3684	4865	4892	5857	5882	162	185
**HPV 33**	Primer-F	1526	1550	2777	2798	3303	3326	4742	4767	5648	5670	122	145
	Primer-R	2492	2518	3654	3673	4213	4233	5656	5678	6491	6514	982	1005
	Probe	1801	1825	2989	3015	3661	3683	4919	4946	5899	5924	163	186
**HPV 35**	Primer-F	1491	1515	2724	2745	3250	3273	4713	4738	5628	5650	123	146
	Primer-R	2440	2469	3640	3659	4187	4207	5636	5658	6474	6497	971	994
	Probe	1751	1775	2936	2962	3647	3669	4890	4917	5876	5901	164	187
**HPV 39**	Primer-F	1614	1638	2868	2889	3397	3420	4806	4831	5724	5746	126	149
	Primer-R	2580	2606	3781	3800	4280	4300	5732	5754	6564	6587	1031	1054
	Probe	1889	1913	3080	3106	3788	3810	4983	5010	5972	5997	167	190
**HPV 41**	Primer-F	1523	1547	2765	2786	3296	3319	4598	4625	5600	5622	146	170
	Primer-R	2483	2509	3726	3745	4118	4138	5608	5630	6476	6499	1075	1098
	Probe	1789	1813	2977	3003	3733	3755	4778	4805	5845	5870	192	215
**HPV 42**	Primer-F	1479	1503	2730	2751	3259	3282	4964	4989	5891	5913	130	153
	Primer-R	2445	2471	3724	3743	4426	4446	5899	5921	6734	6757	929	952
	Probe	1754	1778	2942	2968	3731	3753	5141	5168	6136	6161	171	194
**HPV 45**	Primer-F	1561	1585	2815	2836	3344	3367	4765	4790	5662	5684	121	144
	Primer-R	2527	2553	3725	3744	4239	4259	5670	5692	6514	6537	1017	1040
	Probe	1836	1860	3027	3053	3732	3753	4942	4969	5913	5938	162	185
**HPV 51**	Primer-F	1500	1524	2748	2769	3271	3294	4666	4690	5575	5597	110	133
	Primer-R	2466	2492	3649	3668	4137	4157	5583	5605	6418	6441	974	997
	Probe	1775	1799	2960	2986	3656	3677	4843	4870	5820	5845	151	174
**HPV 52**	Primer-F	1520	1544	2771	2792	3297	3320	4822	4847	5725	5747	115	138
	Primer-R	2486	2512	3690	3709	4293	4313	5733	5755	6583	6606	967	990
	Probe	1795	1819	2983	3009	3697	3721	4999	5026	5982	6007	156	179
**HPV 53**	Primer-F	1519	1543	2769	2790	3295	3318	4812	4837	5709	5731	127	150
	Primer-R	2484	2510	3730	3749	4286	4306	5717	5739	6546	6569	992	1015
	Probe	1793	1817	2981	3007	3737	3759	4989	5016	5951	5976	168	191
**HPV 56**	Primer-F	1524	1548	2775	2796	3301	3324	4752	4777	5652	5674	124	147
	Primer-R	2490	2516	3706	3725	4226	4246	5660	5682	6492	6515	998	1021
	Probe	1799	1823	2987	3013	3713	3735	4929	4956	5897	5922	165	188
**HPV 58**	Primer-F	1530	1554	2781	2802	3307	3330	4776	4801	5697	5719	123	146
	Primer-R	2496	2522	3673	3692	4247	4267	5705	5727	6540	6563	986	1009
	Probe	1805	1829	2993	3019	3680	3702	4953	4980	5948	5973	164	187
**HPV 59**	Primer-F	1522	1546	2776	2797	3305	3328	4757	4782	5660	5682	74	97
	Primer-R	2488	2514	3692	3711	4234	4254	5668	5690	6503	6526	975	998
	Probe	1797	1821	2988	3015	3699	3721	4934	4961	5908	5933	115	138
**HPV 61**	Primer-F	1476	1500	2739	2760	3271	3294	4930	4955	5818	5840	109	132

	Primer-R	2442	2468	3691	3710	4398	4418	5826	5848	6664	6687	929	952
	Probe	1751	1775	2951	2977	3698	3720	5104	5131	6072	6097	147	170
**HPV 62**	Primer-F	1384	1408	2647	2668	3176	3199	4901	4926	5831	5853	14	37
	Primer-R	2350	2376	3596	3615	4363	4383	5839	5861	6677	6700	837	860
	Probe	1659	1683	2859	2885	3603	3625	5087	5114	6079	6104	52	75
**HPV 66**	Primer-F	1506	1630	2757	2778	3283	3306	4801	4826	5701	5723	124	147
	Primer-R	2472	2498	3682	3701	4275	4295	5709	5731	6541	6564	998	1021
	Probe	1781	1805	2969	2995	3689	3711	4978	5005	5946	5971	165	188
**HPV 68**	Primer-F	1462	1486	2716	2737	3245	3268	4654	4679	5569	5591	20	43
	Primer-R	2428	2454	3629	3648	4128	4148	5577	5599	6412	6435	927	950
	Probe	1737	1861	2928	2954	3636	3658	4831	4858	5817	5842	61	84
**HPV 69**	Primer-F	1512	1536	2760	2781	3286	3309	4686	4711	5595	5616	115	138
	Primer-R	2478	2504	3691	3710	4160	4180	5603	5625	6441	6464	986	1009
	Probe	1787	1811	2972	2998	3698	3719	4866	4893	5837	5862	156	179
**HPV 74**	Primer-F	1365	1389	2613	2634	3139	3162	4739	4764	5642	5664	17	40
	Primer-R	2331	2357	3565	3584	4213	4233	5650	5672	6476	6499	818	841
	Probe	1640	1664	2825	2851	3572	3593	4916	4943	5884	5909	58	81
**HPV 93**	Primer-F	1370	1394	2612	2633	3141	3164	4676	4703	5697	5719	136	159
	Primer-R	2327	2353	3601	3620	4074	4094	5705	5727	6579	6602	916	939
	Probe	1633	1657	2824	2850	3608	3630	4888	4915	5945	5970	177	200

* Primer-F, the forward primer of a primer pair. Primer-R, the reverse primer of a primer pair. Probe, the TaqMan probe that was used in combination with the primer pair in a real-time PCR system. The 5' and 3' positions of each primer and probe were given based on the GenBank accession numbers AF092932 for HPV 6, FR872717 for HPV 11, NC_001526 for HPV 16, AY262282 for HPV 18, NC_001583 for HPV 26, J04353 for HPV 31, M12732 for HPV 33, M74117 for HPV 35, KC470244 for HPV 39, NC_001354 for HPV 41, GQ472847 for HPV 42, KC470251 for HPV 45, M62877 for HPV 51, GQ472848 for HPV 52, NC_001593 for HPV 53, EF177181 for HPV 56, FJ385261 for HPV 58, X77858 for HPV 59, U31793 for HPV 61, AY395706 for HPV 62, U31794 for HPV 66, GQ472851 for HPV 68, AB027020 for HPV 69, AF436130 for HPV 74 and AY382778 for HPV 93. For DIPS, each member of the primer pairs and its reversely complementary form were separately used for the 24 independent amplification reactions. For the E2/E6E7 ratio analysis, only the primer pairs and probes corresponding to the E2 and E6E7 gene regions were used. For the E1-L1/E6E7 ratio analysis, the E1-, E2-, E4E5-, L1-, L2- and E6E7-primer pairs and probes were used. Additionally, the primers and probe used for determining the copy number of ACTB gene are as follows: ACTB-F, 5'-agcctcgcctttgccga-3', ACTB-R, 5'-ctggtgcctggggcg-3'and ACTB-P, 5'-FAM-ccgccgcccgtccacacccgcc-TAMRA-3'. For DIPS, the adaptor-specific primer (ASP) used in the exponential amplification stage is 5'-ggccatcagtcagcagtcgtag-3'.

Table S2. Comparison of the diagnostic performances of DIPS, E2/E6E7 ratio analysis and multiple E1-L1/E6E7 ratio analysis in the 25 genotypes of HPVs.*

HPV genotypes	DIPS		E2/E6E7 ratio analysis			E1-L1/E6E7 ratio analysis			κvalue [a§]	κvalue [b§]	κvalue [c§]
	Episomal	Mixed/Integrated	Episomal	Mixed	Integrated	Episomal	Mixed	Integrated			
**HPV 6 (n=27)**	25 (92.6)	2 (7.4)	26 (96.3)	1 (3.7)	0 (0)	26 (96.3)	1 (3.7)	0 (0)	0.649	0.649	1.000
**HPV 11 (n=9)**	9 (100)	0 (0)	9 (100)	0 (0)	0 (0)	9 (100)	0 (0)	0 (0)	N.A.[†]	N.A.	N.A.
**HPV 16 (n=602)**	436 (72.4)	166 (27.6)	480 (79.7)	112 (18.6)	10 (1.7)	448 (74.4)	99 (16.5)	55 (9.1)	0.792	0.932	0.618
**HPV 18 (n=126)**	73 (57.9)	53 (42.1)	87 (69.0)	21 (16.7)	18 (14.3)	71 (56.3)	20 (15.9)	35 (27.8)	0.730	0.968	0.374
**HPV 26 (n=2)**	1 (50.0)	1 (50.0)	1 (50.0)	1 (50.0)	0 (0)	1 (50.0)	1 (50.0)	0 (0)	1.000	1.000	1.000
**HPV 31 (n=79)**	44 (55.7)	35 (44.3)	51 (64.6)	16 (18.9)	12 (16.5)	50 (63.3)	8 (10.1)	21 (26.6)	0.817	0.843	0.785
**HPV 33 (n=89)**	53 (59.6)	36 (40.4)	57 (64.0)	25 (28.1)	7 (7.9)	52 (58.4)	8 (9.0)	29 (32.6)	0.858	0.977	0.570
**HPV 35 (n=29)**	19 (65.5)	10 (34.5)	20 (69.0)	5 (17.2)	4 (13.8)	17 (58.6)	5 (17.2)	7 (24.2)	0.609	0.854	0.806
**HPV 39 (n=35)**	21 (60.0)	14 (40.0)	24 (68.6)	8 (22.9)	3 (8.5)	21 (60.0)	8 (22.9)	6 (17.1)	0.815	1.000	0.836
**HPV 41 (n=3)**	3 (100)	0 (0)	3 (100)	0 (0)	0 (0)	3 (100)	0 (0)	0 (0)	N.A.	N.A.	N.A.
**HPV 42 (n=5)**	4 (80.0)	1 (20.0)	5 (100)	0 (0)	0 (0)	5 (100)	0 (0)	0 (0)	N.A.	N.A.	N.A.
**HPV 45 (n=8)**	4 (50.0)	4 (50.0)	4 (50.0)	4 (50.0)	0 (0)	4 (50.0)	4 (50.0)	0 (0)	1.000	1.000	1.000
**HPV 51 (n=8)**	5 (62.5)	3 (37.5)	5 (62.5)	2 (25.0)	1 (12.5)	5 (62.5)	2 (25.0)	1 (12.5)	1.000	1.000	1.000
**HPV 52 (n=13)**	8 (61.5)	5 (8.5)	10 (76.9)	2 (15.4)	1 (7.7)	10 (76.9)	1 (7.7)	2 (15.4)	0.649	0.649	0.800
**HPV 53 (n=26)**	20 (76.9)	6 (23.1)	22 (84.6)	2 (7.7)	2 (7.7)	22 (84.6)	1 (3.8)	3 (11.5)	0.755	0.755	0.859
**HPV 56 (n=21)**	16 (76.2)	5 (23.8)	16 (76.2)	3 (14.3)	2 (9.5)	16 (76.2)	3 (14.3)	2 (9.5)	1.000	1.000	1.000
**HPV 58 (n=19)**	16 (84.2)	3 (15.8)	17 (89.5)	1 (5.3)	1 (5.3)	16 (84.2)	2 (10.5)	1 (5.3)	0.771	1.000	0.779
**HPV 59 (n=7)**	6 (85.7)	1 (14.3)	6 (85.7)	1 (14.3)	0 (0)	6 (85.7)	1 (14.3)	0 (0)	1.000	1.000	1.000
**HPV 61 (n=5)**	4 (80.0)	1 (20.0)	4 (80.0)	1 (20.0)	0 (0)	4 (80.0)	1 (20.0)	0 (0)	1.000	1.000	1.000
**HPV 62 (n=6)**	5 (83.3)	1 (16.7)	5 (83.3)	1 (16.7)	0 (0)	4 (66.7)	1 (16.7)	1 (16.7)	-0.200	0.571	1.000
**HPV 66 (n=16)**	12 (75.0)	4 (25.0)	12 (75.0)	3 (18.8)	1 (6.3)	12 (75)	3 (18.8)	1 (6.3)	1.000	1.000	N.A.
**HPV 68 (n=18)**	14 (77.8)	4 (22.2)	15 (83.5)	2 (11.1)	1 (5.6)	14 (77.8)	1 (5.5)	3 (16.7)	0.824	1.000	0.670
**HPV 69 (n=7)**	5 (71.4)	2 (28.6)	5 (71.4)	1 (14.3)	1 (14.3)	5 (71.4)	0 (0)	2 (28.6)	1.000	1.000	N.A.
**HPV 74 (n=1)**	1 (100)	0 (0)	1 (100)	0 (0)	0 (0)	1 (100)	0 (0)	0 (0)	N.A.	N.A.	N.A.
**HPV 93 (n=1)**	1 (100)	0 (0)	1 (100)	0 (0)	0 (0)	1 (100)	0 (0)	0 (0)	N.A.	N.A.	N.A.

* Data are presented as number (%).

a The detection consistency was compared between DIPS and E2/E6E7 ratio analysis using Cohen's κ value. We defined the κ value of 0–0.20 as slight, 0.21–0.40 as fair, 0.41–0.60 as moderate, 0.61–0.80 as substantial, and 0.81–1 as perfect consistency between two series of detection data. To accommodate the DIPS system, which only provides information on whether or not the integration event has happened, we merged the mixed and integrated infection statuses detected by E2/E6E7 ratio analysis into one category, that is, the mixed/integrated infection status, to perform the κ value calculation.

b The detection consistency was compared between DIPS and E1-L1/E6E7 ratio analysis using Cohen's κ value. The mixed HPV infection and integrated HPV infection detected by E2/E6E7 ratio analysis were merged into a mixed/integrated infection status to perform the κ value calculation.

c The detection consistency was compared between E2/E6E7 ratio analysis and E1-L1/E6E7 ratio analysis.
§ The detailed data used for calculating the κ value are listed in Table S6-S30.
†N.A., not available. The κ value could not be calculated because one or both of the compared data series were constants.

Table S3. Detection consistency between DIPS and E2/E6E7 ratio analysis in the enrolled 1162 women.*

Items		E2/E6E7 ratio analysis	
		Episomal	Mixed/Integrated
DIPS	Episomal	799	6
	Mixed/Integrated	87	270
κ value		0.799	

* The detection consistency was compared between DIPS and E2/E6E7 ratio analysis using Cohen's κ value. We defined the κ value of 0–0.20 as slight, 0.21–0.40 as fair, 0.41–0.60 as moderate, 0.61–0.80 as substantial, and 0.81–1 as perfect consistency between two series of detection data.

Table S4. Detection consistency between DIPS and multiple E1-L1/E6E7 ratio analysis in the enrolled 1162 women.*

Items		E1-L1/E6E7 ratio analysis	
		Episomal	Mixed/Integrated
DIPS	Episomal	797	8
	Mixed/Integrated	26	331
κ value		0.930	

* The detection consistency was compared between DIPS and E1-L1/E6E7 ratio analysis using Cohen's κ value. We defined the κ value of 0–0.20 as slight, 0.21–0.40 as fair, 0.41–0.60 as moderate, 0.61–0.80 as substantial, and 0.81–1 as perfect consistency between two series of detection data.

Table S5. Detection consistency between E2/E6E7 ratio analysis and multiple E1-L1/E6E7 ratio analysis in the enrolled 1162 women.*

Items		E1-L1/E6E7 ratio analysis		
		Episomal	Mixed	Integrated
E2/E6E7 ratio analysis	Episomal	823	1	62
	Mixed	0	112	100
	Integrated	0	13	51
κ value		0.648**		

* The detection consistency was compared between E2/E6E7 ratio analysis and E1-L1/E6E7 ratio analysis using Cohen's κ value. We defined the κ value of 0–0.20 as slight, 0.21–0.40 as fair, 0.41–0.60 as moderate, 0.61–0.80 as substantial, and 0.81–1 as perfect consistency between two series of detection data.
** If the mixed HPV infection and integrated HPV infection are taken as a whole, the κ value is 0.861 between E2/E6E7 ratio analysis and E1-L1/E6E7 ratio analysis.

Table S6. Detection results of DIPS, E2/E6E7 ratio analysis and multiple E1-L1/E6E7 ratio analysis in the HPV 6-positive cases.

Patient No.	DIPS	E2/E6E7 ratio analysis	E1-L1/E6E7 ratio analysis
102370	Negative	Negative	Negative
119395	Negative	Negative	Negative
130000	Negative	Negative	Negative
135157	Mixed/Integrated	Negative	Negative
137892	Negative	Negative	Negative
138025	Negative	Negative	Negative
143837	Negative	Negative	Negative
145414	Negative	Negative	Negative

147441	Negative	Negative	Negative
147728	Negative	Negative	Negative
149377	Negative	Negative	Negative
150484	Negative	Negative	Negative
151119	Negative	Negative	Negative
151225	Negative	Negative	Negative
152179	Negative	Negative	Negative
197769	Negative	Negative	Negative
198132	Negative	Negative	Negative
497444	Negative	Negative	Negative
513110	Negative	Negative	Negative
518332	Negative	Negative	Negative
594031	Negative	Negative	Negative
611980	Negative	Negative	Negative
629931	Negative	Negative	Negative
636457	Negative	Negative	Negative
638090	Negative	Negative	Negative
640794	Mixed/Integrated	Mixed	Mixed
648697	Negative	Negative	Negative

Table S7. Detection results of DIPS, E2/E6E7 ratio analysis and multiple E1-L1/E6E7 ratio analysis in the HPV 11-positive cases.

Patient No.	DIPS	E2/E6E7 ratio analysis	E1-L1/E6E7 ratio analysis
138943	Negative	Negative	Negative
141907	Negative	Negative	Negative
144079	Negative	Negative	Negative
151778	Negative	Negative	Negative
153016	Negative	Negative	Negative
180930	Negative	Negative	Negative
211061	Negative	Negative	Negative
138943	Negative	Negative	Negative
141907	Negative	Negative	Negative

Table S8. Detection results of DIPS, E2/E6E7 ratio analysis and multiple E1-L1/E6E7 ratio analysis in the HPV 16-positive cases.

Patient No.	DIPS	E2/E6E7 ratio analysis	E1-L1/E6E7 ratio analysis
126959	Negative	Negative	Negative
138671	Negative	Negative	Negative
100236	Negative	Negative	Negative
100369	Negative	Negative	Negative
100556	Negative	Negative	Negative
100877	Negative	Negative	Negative
101080	Negative	Negative	Negative
101269	Mixed/Integrated	Mixed	Integrated
101484	Mixed/Integrated	Mixed	Integrated
102147	Negative	Negative	Negative

103748	Negative	Negative	Negative
104732	Mixed/Integrated	Mixed	Integrated
105349	Negative	Negative	Negative
106584	Negative	Negative	Negative
106585	Negative	Negative	Negative
107305	Negative	Negative	Negative
107483	Negative	Negative	Negative
107497	Negative	Negative	Negative
107598	Negative	Negative	Negative
107599	Negative	Negative	Negative
107600	Negative	Negative	Negative
108240	Negative	Negative	Negative
108907	Negative	Negative	Negative
109518	Negative	Negative	Negative
109520	Negative	Negative	Negative
109762	Negative	Negative	Negative
109854	Negative	Negative	Negative
110907	Mixed/Integrated	Negative	Integrated
111041	Negative	Negative	Negative
111156	Negative	Negative	Negative
111396	Negative	Negative	Negative
111400	Mixed/Integrated	Mixed	Integrated
111930	Negative	Negative	Negative
112464	Negative	Negative	Negative
113353	Negative	Negative	Negative
114954	Negative	Negative	Negative
115145	Mixed/Integrated	Mixed	Integrated
115284	Negative	Negative	Negative
115732	Negative	Negative	Negative
115843	Negative	Negative	Negative
115885	Negative	Negative	Negative
116199	Negative	Negative	Negative
116785	Negative	Negative	Negative
116786	Negative	Negative	Negative
117087	Negative	Negative	Negative
117800	Negative	Negative	Negative
118067	Mixed/Integrated	Mixed	Integrated
118240	Negative	Negative	Negative
118286	Negative	Negative	Negative
118335	Negative	Negative	Negative
118689	Negative	Negative	Negative
118887	Negative	Negative	Negative
119045	Negative	Negative	Negative
119936	Negative	Negative	Negative
120303	Negative	Negative	Negative
120824	Mixed/Integrated	Mixed	Integrated
121011	Negative	Negative	Negative
121076	Negative	Negative	Negative
121077	Negative	Negative	Negative

121265	Negative	Negative	Negative
121272	Negative	Negative	Negative
121299	Mixed/Integrated	Mixed	Integrated
121532	Negative	Negative	Negative
121664	Negative	Negative	Negative
122603	Negative	Negative	Negative
122604	Negative	Negative	Negative
122834	Negative	Negative	Negative
123020	Negative	Negative	Negative
123297	Negative	Negative	Negative
123314	Negative	Negative	Negative
123590	Negative	Negative	Negative
123649	Negative	Negative	Negative
123650	Negative	Negative	Negative
123650	Negative	Negative	Negative
123687	Negative	Negative	Negative
123689	Negative	Negative	Negative
123811	Mixed/Integrated	Mixed	Integrated
124866	Mixed/Integrated	Mixed	Integrated
124878	Mixed/Integrated	Mixed	Integrated
125315	Negative	Negative	Negative
125322	Negative	Negative	Negative
125583	Negative	Negative	Negative
126450	Negative	Negative	Negative
126851	Negative	Negative	Negative
126938	Negative	Negative	Negative
126986	Negative	Negative	Negative
127201	Negative	Negative	Negative
127274	Negative	Negative	Negative
127454	Mixed/Integrated	Mixed	Integrated
128141	Negative	Negative	Negative
128710	Negative	Negative	Negative
128711	Negative	Negative	Negative
128845	Negative	Negative	Negative
128848	Negative	Negative	Negative
129205	Negative	Negative	Negative
129207	Negative	Negative	Negative
129208	Negative	Negative	Negative
129213	Negative	Negative	Negative
129440	Negative	Negative	Negative
129534	Negative	Negative	Negative
129813	Negative	Negative	Negative
130217	Negative	Negative	Negative
130529	Negative	Negative	Negative
130540	Negative	Negative	Negative
130630	Negative	Negative	Negative
130899	Negative	Negative	Negative
131362	Negative	Negative	Negative
131387	Negative	Negative	Negative

131413	Negative	Negative	Negative
131417	Negative	Negative	Negative
131493	Negative	Negative	Negative
131592	Negative	Negative	Negative
131594	Mixed/Integrated	Mixed	Mixed
131829	Negative	Negative	Negative
131885	Negative	Negative	Negative
132069	Negative	Negative	Negative
132477	Negative	Negative	Negative
132506	Mixed/Integrated	Mixed	Mixed
133135	Mixed/Integrated	Mixed	Mixed
133159	Negative	Negative	Negative
133189	Negative	Negative	Negative
133198	Negative	Negative	Negative
133290	Negative	Negative	Negative
133625	Negative	Negative	Negative
133626	Mixed/Integrated	Mixed	Integrated
133646	Negative	Negative	Negative
133683	Negative	Negative	Negative
133818	Negative	Negative	Negative
133922	Negative	Negative	Negative
134250	Negative	Negative	Negative
134315	Negative	Negative	Negative
134355	Negative	Negative	Negative
134358	Negative	Negative	Negative
134494	Negative	Negative	Negative
134507	Negative	Negative	Negative
134615	Negative	Negative	Negative
134908	Negative	Negative	Negative
134915	Negative	Negative	Negative
134923	Negative	Negative	Negative
134928	Negative	Negative	Negative
135020	Mixed/Integrated	Mixed	Mixed
135059	Negative	Negative	Negative
135275	Negative	Negative	Negative
135280	Negative	Negative	Negative
135838	Negative	Negative	Negative
135895	Negative	Negative	Negative
135898	Negative	Negative	Negative
136158	Negative	Negative	Negative
136188	Negative	Negative	Negative
136255	Negative	Negative	Negative
136711	Negative	Negative	Negative
136781	Negative	Negative	Negative
137073	Negative	Negative	Negative
137079	Mixed/Integrated	Mixed	Mixed
137318	Negative	Negative	Negative
137422	Mixed/Integrated	Mixed	Integrated
137481	Negative	Negative	Negative

137584	Negative	Negative	Negative
137984	Negative	Negative	Negative
138159	Negative	Negative	Negative
138402	Negative	Negative	Negative
138468	Negative	Negative	Negative
138485	Negative	Negative	Negative
138496	Negative	Negative	Negative
138686	Negative	Negative	Negative
138725	Negative	Negative	Negative
138783	Negative	Negative	Negative
139289	Negative	Negative	Negative
139291	Negative	Negative	Negative
139584	Negative	Negative	Negative
139621	Negative	Negative	Negative
139684	Negative	Negative	Negative
139784	Negative	Negative	Negative
139973	Mixed/Integrated	Mixed	Mixed
140076	Mixed/Integrated	Mixed	Integrated
140163	Mixed/Integrated	Negative	Integrated
140203	Negative	Negative	Negative
140276	Negative	Negative	Negative
140278	Mixed/Integrated	Mixed	Mixed
140284	Negative	Negative	Negative
140644	Negative	Negative	Negative
140846	Negative	Negative	Negative
140855	Mixed/Integrated	Mixed	Integrated
141122	Mixed/Integrated	Mixed	Integrated
141189	Negative	Negative	Negative
141455	Mixed/Integrated	Mixed	Integrated
141531	Negative	Negative	Negative
141536	Mixed/Integrated	Mixed	Mixed
141654	Negative	Negative	Negative
141666	Negative	Negative	Negative
141676	Mixed/Integrated	Mixed	Mixed
141684	Negative	Negative	Negative
141695	Mixed/Integrated	Mixed	Integrated
142110	Mixed/Integrated	Mixed	Mixed
142185	Negative	Negative	Negative
142187	Mixed/Integrated	Negative	Negative
142189	Negative	Negative	Negative
142190	Mixed/Integrated	Mixed	Integrated
142219	Negative	Negative	Negative
142387	Negative	Negative	Negative
143020	Mixed/Integrated	Negative	Integrated
143326	Mixed/Integrated	Mixed	Mixed
143435	Negative	Negative	Negative
143436	Negative	Negative	Negative
143439	Negative	Negative	Negative
143439	Negative	Negative	Negative

143440	Negative	Negative	Negative
143441	Negative	Negative	Negative
143444	Mixed/Integrated	Mixed	Integrated
143573	Negative	Negative	Negative
143936	Mixed/Integrated	Mixed	Mixed
144292	Negative	Negative	Negative
144594	Negative	Negative	Negative
144594	Negative	Negative	Negative
144691	Mixed/Integrated	Integrated	Integrated
145305	Negative	Negative	Negative
145545	Negative	Negative	Negative
145762	Negative	Negative	Negative
145789	Mixed/Integrated	Mixed	Integrated
145919	Negative	Negative	Negative
146359	Negative	Negative	Negative
146374	Mixed/Integrated	Mixed	Mixed
146453	Negative	Negative	Negative
146526	Negative	Negative	Negative
146551	Mixed/Integrated	Mixed	Mixed
146551	Mixed/Integrated	Mixed	Mixed
146586	Negative	Negative	Negative
146982	Negative	Negative	Negative
147094	Negative	Negative	Negative
147097	Negative	Negative	Negative
147097	Mixed/Integrated	Mixed	Integrated
147204	Negative	Negative	Negative
147524	Mixed/Integrated	Mixed	Integrated
147575	Negative	Negative	Negative
147592	Negative	Negative	Negative
147686	Mixed/Integrated	Negative	Integrated
148048	Negative	Negative	Negative
148055	Negative	Negative	Negative
148242	Negative	Negative	Negative
148802	Negative	Negative	Negative
149015	Negative	Negative	Negative
149049	Negative	Negative	Negative
149259	Negative	Negative	Negative
150147	Negative	Negative	Negative
150720	Mixed/Integrated	Mixed	Mixed
150790	Negative	Negative	Negative
151023	Negative	Negative	Negative
151111	Negative	Negative	Negative
151247	Negative	Negative	Negative
151251	Mixed/Integrated	Mixed	Mixed
151255	Mixed/Integrated	Mixed	Integrated
151411	Negative	Negative	Negative
151504	Mixed/Integrated	Mixed	Integrated
151511	Negative	Negative	Negative
151645	Mixed/Integrated	Mixed	Mixed

151687	Mixed/Integrated	Negative	Integrated
151984	Negative	Negative	Negative
152052	Negative	Negative	Negative
152075	Negative	Negative	Negative
152078	Mixed/Integrated	Mixed	Integrated
152146	Negative	Negative	Negative
152421	Negative	Negative	Negative
152496	Negative	Negative	Negative
152610	Negative	Negative	Negative
152621	Negative	Negative	Negative
152624	Mixed/Integrated	Mixed	Mixed
152856	Mixed/Integrated	Mixed	Integrated
152857	Negative	Negative	Negative
152858	Mixed/Integrated	Mixed	Integrated
152931	Negative	Negative	Negative
153170	Negative	Negative	Negative
153278	Mixed/Integrated	Mixed	Mixed
153294	Negative	Negative	Negative
153339	Mixed/Integrated	Mixed	Integrated
153688	Mixed/Integrated	Integrated	Integrated
153692	Mixed/Integrated	Integrated	Integrated
153782	Negative	Negative	Negative
153816	Negative	Negative	Negative
153818	Negative	Negative	Negative
154009	Negative	Negative	Negative
154100	Mixed/Integrated	Negative	Integrated
154217	Negative	Negative	Negative
154466	Negative	Negative	Negative
154744	Negative	Negative	Negative
155194	Negative	Negative	Negative
155363	Mixed/Integrated	Negative	Integrated
155382	Negative	Negative	Negative
155523	Mixed/Integrated	Mixed	Integrated
155678	Negative	Negative	Negative
155689	Mixed/Integrated	Integrated	Integrated
155822	Mixed/Integrated	Mixed	Integrated
156049	Negative	Negative	Negative
156144	Negative	Negative	Negative
156231	Negative	Negative	Negative
156251	Negative	Negative	Negative
156274	Negative	Negative	Negative
156282	Mixed/Integrated	Mixed	Mixed
156526	Negative	Negative	Negative
156544	Mixed/Integrated	Mixed	Mixed
156827	Negative	Negative	Negative
156829	Mixed/Integrated	Mixed	Mixed
156859	Negative	Negative	Negative
157053	Negative	Negative	Negative
157223	Mixed/Integrated	Negative	Negative

157482	Mixed/Integrated	Mixed	Mixed
157658	Negative	Negative	Negative
157658	Negative	Negative	Negative
157790	Negative	Negative	Negative
157791	Mixed/Integrated	Mixed	Integrated
157945	Mixed/Integrated	Mixed	Integrated
158089	Mixed/Integrated	Negative	Integrated
158107	Negative	Negative	Negative
158175	Negative	Negative	Negative
158176	Mixed/Integrated	Negative	Integrated
158239	Negative	Negative	Negative
158335	Negative	Negative	Negative
158584	Negative	Negative	Negative
158890	Mixed/Integrated	Integrated	Integrated
159021	Negative	Negative	Negative
159316	Negative	Negative	Negative
159317	Negative	Negative	Negative
159404	Negative	Negative	Negative
159727	Negative	Negative	Negative
160619	Negative	Negative	Negative
160622	Negative	Negative	Negative
160860	Negative	Negative	Negative
161192	Mixed/Integrated	Negative	Integrated
161212	Mixed/Integrated	Mixed	Integrated
161213	Mixed/Integrated	Mixed	Integrated
161299	Negative	Negative	Negative
161497	Mixed/Integrated	Negative	Negative
161587	Negative	Negative	Negative
161660	Negative	Negative	Negative
161894	Mixed/Integrated	Mixed	Mixed
161899	Negative	Negative	Negative
161899	Negative	Negative	Negative
161901	Negative	Negative	Negative
162153	Negative	Negative	Negative
162211	Negative	Negative	Negative
162700	Negative	Negative	Negative
162746	Mixed/Integrated	Mixed	Integrated
162778	Mixed/Integrated	Mixed	Mixed
162890	Mixed/Integrated	Negative	Integrated
163141	Mixed/Integrated	Mixed	Integrated
163615	Negative	Negative	Negative
163691	Mixed/Integrated	Integrated	Integrated
163817	Negative	Negative	Negative
163988	Negative	Negative	Negative
164058	Mixed/Integrated	Mixed	Mixed
164164	Negative	Negative	Negative
164166	Mixed/Integrated	Mixed	Integrated
164861	Negative	Negative	Negative
164888	Negative	Negative	Negative

165781	Negative	Negative	Negative
165995	Negative	Negative	Negative
166333	Negative	Negative	Negative
166480	Mixed/Integrated	Mixed	Integrated
166635	Negative	Negative	Negative
167054	Negative	Negative	Negative
167056	Mixed/Integrated	Mixed	Mixed
167245	Negative	Negative	Negative
167249	Negative	Negative	Negative
167251	Negative	Negative	Negative
167253	Mixed/Integrated	Mixed	Mixed
167263	Negative	Negative	Negative
167550	Mixed/Integrated	Negative	Negative
167593	Negative	Negative	Negative
167595	Mixed/Integrated	Mixed	Mixed
167691	Mixed/Integrated	Negative	Integrated
167898	Mixed/Integrated	Negative	Negative
168002	Negative	Negative	Negative
168074	Negative	Negative	Negative
168455	Mixed/Integrated	Mixed	Mixed
168456	Mixed/Integrated	Negative	Integrated
168456	Mixed/Integrated	Mixed	Mixed
168499	Negative	Negative	Negative
168556	Negative	Negative	Negative
168612	Negative	Negative	Negative
168862	Negative	Negative	Negative
168930	Negative	Negative	Negative
169389	Negative	Negative	Negative
169389	Negative	Negative	Integrated
169390	Mixed/Integrated	Mixed	Mixed
169410	Negative	Negative	Negative
169766	Negative	Negative	Negative
169769	Mixed/Integrated	Mixed	Mixed
169849	Negative	Negative	Negative
170279	Mixed/Integrated	Mixed	Mixed
170280	Mixed/Integrated	Mixed	Integrated
170463	Negative	Negative	Negative
170463	Negative	Negative	Negative
170465	Negative	Negative	Negative
170961	Mixed/Integrated	Mixed	Integrated
171173	Mixed/Integrated	Mixed	Mixed
171280	Negative	Negative	Negative
171292	Mixed/Integrated	Negative	Integrated
171326	Negative	Negative	Negative
171368	Mixed/Integrated	Negative	Integrated
171544	Negative	Negative	Negative
171692	Mixed/Integrated	Negative	Integrated
172278	Mixed/Integrated	Mixed	Mixed
172803	Negative	Negative	Negative

172890	Negative	Negative	Negative
173038	Negative	Negative	Negative
173172	Negative	Negative	Negative
173369	Mixed/Integrated	Negative	Negative
173946	Negative	Negative	Negative
174931	Negative	Negative	Negative
175683	Negative	Negative	Negative
175883	Negative	Negative	Negative
176483	Negative	Negative	Negative
176858	Mixed/Integrated	Mixed	Mixed
177149	Mixed/Integrated	Negative	Integrated
177389	Negative	Negative	Negative
177391	Mixed/Integrated	Mixed	Mixed
177484	Negative	Negative	Negative
177657	Negative	Negative	Negative
177896	Negative	Negative	Negative
178083	Negative	Negative	Negative
178239	Negative	Negative	Negative
178284	Negative	Negative	Negative
178452	Negative	Negative	Negative
178484	Negative	Negative	Negative
178684	Negative	Negative	Negative
179189	Mixed/Integrated	Negative	Integrated
179275	Negative	Mixed	Mixed
179276	Negative	Negative	Negative
179281	Negative	Negative	Negative
179282	Negative	Negative	Negative
179284	Mixed/Integrated	Mixed	Integrated
179285	Mixed/Integrated	Mixed	Integrated
179855	Negative	Negative	Negative
180277	Negative	Negative	Negative
180457	Mixed/Integrated	Negative	Integrated
180685	Negative	Negative	Negative
180872	Negative	Negative	Negative
180885	Negative	Negative	Negative
180982	Negative	Negative	Negative
180983	Negative	Negative	Negative
180985	Mixed/Integrated	Mixed	Mixed
181088	Mixed/Integrated	Negative	Integrated
181421	Mixed/Integrated	Mixed	Mixed
181426	Negative	Negative	Negative
181657	Negative	Negative	Negative
181661	Negative	Negative	Negative
181933	Mixed/Integrated	Negative	Integrated
181996	Mixed/Integrated	Mixed	Mixed
182012	Negative	Negative	Negative
182191	Mixed/Integrated	Negative	Integrated
182479	Negative	Negative	Negative
182480	Negative	Negative	Negative

182482	Negative	Negative	Negative
182486	Negative	Negative	Negative
182487	Negative	Negative	Negative
182488	Mixed/Integrated	Mixed	Integrated
182688	Mixed/Integrated	Negative	Integrated
182718	Negative	Negative	Negative
182798	Mixed/Integrated	Mixed	Mixed
183084	Negative	Negative	Negative
183255	Negative	Negative	Negative
183256	Mixed/Integrated	Mixed	Integrated
183264	Mixed/Integrated	Negative	Integrated
184287	Negative	Negative	Negative
184287	Negative	Negative	Negative
184325	Mixed/Integrated	Mixed	Mixed
184615	Negative	Negative	Negative
184689	Mixed/Integrated	Negative	Integrated
185150	Negative	Negative	Negative
185173	Negative	Negative	Negative
185658	Negative	Negative	Negative
185696	Negative	Negative	Negative
185927	Mixed/Integrated	Negative	Negative
186474	Mixed/Integrated	Mixed	Mixed
186489	Negative	Negative	Negative
186996	Negative	Negative	Negative
187056	Negative	Negative	Negative
187244	Negative	Negative	Negative
187525	Mixed/Integrated	Mixed	Mixed
188689	Negative	Negative	Negative
188876	Mixed/Integrated	Mixed	Integrated
188939	Negative	Negative	Negative
189587	Negative	Negative	Negative
189646	Negative	Negative	Negative
189649	Mixed/Integrated	Mixed	Mixed
190069	Negative	Negative	Negative
190158	Negative	Negative	Negative
190472	Mixed/Integrated	Mixed	Integrated
190731	Negative	Negative	Negative
190953	Negative	Negative	Negative
191012	Negative	Negative	Negative
191636	Mixed/Integrated	Mixed	Integrated
192331	Negative	Negative	Negative
192424	Negative	Negative	Negative
193053	Mixed/Integrated	Mixed	Mixed
193672	Negative	Negative	Negative
193834	Negative	Negative	Negative
193856	Negative	Negative	Negative
193856	Negative	Negative	Negative
193932	Mixed/Integrated	Mixed	Integrated
194475	Negative	Negative	Negative

194701	Negative	Negative	Negative
195082	Negative	Negative	Negative
195115	Negative	Negative	Negative
196560	Mixed/Integrated	Negative	Integrated
196716	Negative	Negative	Negative
196876	Mixed/Integrated	Mixed	Integrated
197676	Negative	Negative	Negative
198065	Mixed/Integrated	Mixed	Integrated
198333	Negative	Negative	Negative
198535	Mixed/Integrated	Negative	Negative
199478	Mixed/Integrated	Negative	Integrated
199700	Mixed/Integrated	Mixed	Integrated
199916	Negative	Negative	Negative
199917	Negative	Negative	Negative
199918	Negative	Negative	Negative
200386	Mixed/Integrated	Integrated	Integrated
200588	Negative	Negative	Negative
201089	Negative	Negative	Negative
207039	Negative	Negative	Negative
208044	Negative	Negative	Negative
209686	Mixed/Integrated	Negative	Integrated
210335	Mixed/Integrated	Mixed	Mixed
211590	Negative	Negative	Negative
211591	Negative	Negative	Negative
212294	Mixed/Integrated	Mixed	Mixed
213270	Negative	Negative	Negative
213687	Mixed/Integrated	Mixed	Integrated
214682	Negative	Negative	Negative
215122	Negative	Negative	Negative
216288	Negative	Negative	Negative
217884	Negative	Negative	Negative
218962	Negative	Negative	Negative
219391	Negative	Negative	Negative
219688	Mixed/Integrated	Mixed	Integrated
220200	Negative	Negative	Negative
221523	Negative	Negative	Negative
222685	Negative	Negative	Negative
223754	Negative	Negative	Negative
224098	Negative	Negative	Negative
226550	Negative	Negative	Negative
229702	Mixed/Integrated	Negative	Integrated
229745	Negative	Negative	Negative
229762	Negative	Negative	Negative
231627	Negative	Negative	Negative
232535	Negative	Negative	Negative
233040	Negative	Negative	Negative
233510	Negative	Negative	Negative
233513	Negative	Negative	Negative
233787	Negative	Negative	Negative

233981	Negative	Negative	Negative
234451	Negative	Negative	Negative
236805	Negative	Negative	Negative
239628	Negative	Negative	Negative
241040	Negative	Negative	Negative
243091	Mixed/Integrated	Negative	Negative
243866	Negative	Negative	Negative
246016	Negative	Negative	Negative
246353	Negative	Negative	Negative
246873	Negative	Negative	Negative
247291	Mixed/Integrated	Negative	Negative
248374	Negative	Negative	Negative
248491	Mixed/Integrated	Negative	Negative
250772	Negative	Negative	Negative
251407	Negative	Negative	Negative
255093	Negative	Negative	Negative
257134	Mixed/Integrated	Negative	Negative
259293	Negative	Negative	Negative
259869	Negative	Negative	Negative
260166	Mixed/Integrated	Negative	Negative
262683	Negative	Negative	Negative
262685	Negative	Negative	Negative
263199	Negative	Negative	Negative
267242	Negative	Negative	Negative
268334	Mixed/Integrated	Negative	Integrated
268899	Negative	Negative	Negative
268902	Mixed/Integrated	Negative	Negative
273984	Mixed/Integrated	Integrated	Integrated
276341	Negative	Negative	Negative
276677	Negative	Negative	Negative
279373	Negative	Negative	Negative
279379	Negative	Negative	Negative
279626	Mixed/Integrated	Integrated	Integrated
279628	Negative	Negative	Negative
280761	Mixed/Integrated	Integrated	Integrated
281722	Negative	Negative	Negative
282742	Negative	Negative	Negative
283225	Mixed/Integrated	Negative	Integrated
286805	Mixed/Integrated	Mixed	Mixed
286807	Mixed/Integrated	Mixed	Mixed
286808	Mixed/Integrated	Mixed	Mixed
290780	Negative	Negative	Negative
291294	Negative	Negative	Negative
291927	Mixed/Integrated	Mixed	Integrated
296894	Negative	Negative	Negative
307869	Negative	Negative	Negative
312388	Negative	Negative	Negative
314082	Negative	Negative	Negative
314646	Negative	Negative	Negative

321703	Negative	Negative	Negative
322645	Mixed/Integrated	Mixed	Integrated
328407	Mixed/Integrated	Mixed	Mixed
343287	Negative	Negative	Negative
355849	Negative	Negative	Negative
358490	Negative	Negative	Negative

Table S9. Detection results of DIPS, E2/E6E7 ratio analysis and multiple E1-L1/E6E7 ratio analysis in the HPV 18-positive cases.

Patient No.	DIPS	E2/E6E7 ratio analysis	E1-L1/E6E7 ratio analysis
112326	Negative	Negative	Negative
112400	Mixed/Integrated	Negative	Integrated
112760	Mixed/Integrated	Negative	Integrated
113318	Mixed/Integrated	Integrated	Mixed
115430	Mixed/Integrated	Integrated	Mixed
116898	Negative	Negative	Negative
121041	Negative	Negative	Negative
121044	Mixed/Integrated	Mixed	Mixed
121762	Negative	Negative	Negative
122712	Mixed/Integrated	Negative	Integrated
123202	Negative	Negative	Negative
123320	Negative	Negative	Negative
123463	Mixed/Integrated	Mixed	Mixed
124214	Mixed/Integrated	Integrated	Mixed
124288	Mixed/Integrated	Integrated	Mixed
125125	Negative	Negative	Negative
125250	Mixed/Integrated	Integrated	Integrated
125712	Negative	Negative	Negative
125933	Negative	Negative	Negative
126206	Negative	Negative	Negative
126207	Mixed/Integrated	Integrated	Integrated
126510	Negative	Negative	Negative
132385	Mixed/Integrated	Integrated	Mixed
136877	Negative	Negative	Negative
137637	Negative	Negative	Negative
137884	Negative	Negative	Negative
138007	Mixed/Integrated	Mixed	Integrated
144965	Negative	Negative	Negative
144966	Mixed/Integrated	Mixed	Integrated
145357	Negative	Negative	Negative
145652	Negative	Negative	Negative
145656	Mixed/Integrated	Mixed	Mixed
147456	Mixed/Integrated	Negative	Integrated
148599	Negative	Negative	Negative
150656	Negative	Negative	Negative
151787	Negative	Negative	Negative
151823	Negative	Negative	Negative
151825	Mixed/Integrated	Mixed	Integrated

153539	Negative	Negative	Integrated
153540	Mixed/Integrated	Mixed	Integrated
154051	Negative	Negative	Negative
154052	Mixed/Integrated	Mixed	Integrated
158216	Negative	Negative	Negative
159370	Mixed/Integrated	Mixed	Mixed
159470	Mixed/Integrated	Negative	Integrated
160755	Negative	Negative	Negative
161034	Mixed/Integrated	Mixed	Integrated
161250	Negative	Negative	Negative
161252	Mixed/Integrated	Mixed	Integrated
161362	Negative	Negative	Negative
161363	Negative	Negative	Negative
162279	Negative	Negative	Negative
162280	Mixed/Integrated	Negative	Integrated
162455	Negative	Negative	Negative
163821	Negative	Negative	Negative
163930	Mixed/Integrated	Mixed	Integrated
164181	Mixed/Integrated	Mixed	Integrated
164646	Negative	Negative	Negative
165413	Mixed/Integrated	Negative	Integrated
165627	Mixed/Integrated	Mixed	Integrated
166729	Mixed/Integrated	Negative	Integrated
166913	Mixed/Integrated	Mixed	Integrated
171076	Negative	Negative	Negative
175657	Mixed/Integrated	Mixed	Integrated
175662	Mixed/Integrated	Integrated	Mixed
176038	Negative	Negative	Negative
176490	Negative	Negative	Negative
177327	Negative	Negative	Negative
177954	Mixed/Integrated	Integrated	Mixed
179056	Negative	Negative	Negative
179264	Mixed/Integrated	Integrated	Integrated
180062	Negative	Negative	Negative
181318	Negative	Negative	Negative
181441	Negative	Negative	Negative
181484	Mixed/Integrated	Negative	Integrated
182130	Negative	Negative	Negative
182888	Mixed/Integrated	Integrated	Mixed
183242	Negative	Negative	Negative
184234	Negative	Negative	Negative
185807	Negative	Negative	Negative
187243	Mixed/Integrated	Negative	Integrated
187611	Negative	Negative	Negative
188965	Mixed/Integrated	Integrated	Mixed
189081	Negative	Negative	Negative
189159	Mixed/Integrated	Mixed	Integrated
189187	Negative	Negative	Negative
189543	Negative	Integrated	Integrated

189835	Mixed/Integrated	Mixed	Mixed
190259	Negative	Negative	Negative
190509	Mixed/Integrated	Integrated	Integrated
190665	Negative	Negative	Negative
191010	Negative	Negative	Negative
191101	Mixed/Integrated	Mixed	Mixed
191598	Negative	Negative	Negative
192353	Negative	Negative	Negative
194563	Mixed/Integrated	Mixed	Mixed
194960	Mixed/Integrated	Integrated	Mixed
195843	Negative	Negative	Negative
196293	Negative	Negative	Negative
196743	Negative	Negative	Negative
198818	Mixed/Integrated	Integrated	Mixed
199064	Mixed/Integrated	Integrated	Mixed
200794	Negative	Negative	Negative
213200	Negative	Negative	Negative
214912	Mixed/Integrated	Mixed	Integrated
219682	Negative	Negative	Negative
223403	Negative	Negative	Negative
224721	Negative	Negative	Negative
226779	Negative	Negative	Negative
229030	Mixed/Integrated	Negative	Integrated
239126	Mixed/Integrated	Integrated	Mixed
239391	Negative	Negative	Negative
245113	Mixed/Integrated	Negative	Integrated
245216	Negative	Negative	Negative
246326	Mixed/Integrated	Negative	Integrated
253159	Negative	Negative	Negative
262160	Negative	Negative	Negative
274144	Negative	Negative	Negative
274144	Negative	Negative	Negative
274818	Negative	Negative	Negative
276169	Negative	Negative	Negative
278193	Negative	Negative	Negative
388898	Mixed/Integrated	Negative	Integrated
441548	Negative	Negative	Negative

Table S10. Detection results of DIPS, E2/E6E7 ratio analysis and multiple E1-L1/E6E7 ratio analysis in the HPV 26-positive cases.

Patient No.	DIPS	E2/E6E7 ratio analysis	E1-L1/E6E7 ratio analysis
111213	Negative	Negative	Negative
214834	Mixed/Integrated	Mixed	Mixed

Table S11. Detection results of DIPS, E2/E6E7 ratio analysis and multiple E1-L1/E6E7 ratio analysis in the HPV 31-positive cases.

Patient No.	DIPS	E2/E6E7 ratio analysis	E1-L1/E6E7 ratio

			analysis
111516	Negative	Negative	Negative
148091	Negative	Negative	Negative
100154	Mixed/Integrated	Mixed	Mixed
104582	Mixed/Integrated	Integrated	Integrated
109010	Mixed/Integrated	Integrated	Integrated
112678	Negative	Negative	Negative
123028	Negative	Negative	Negative
124686	Negative	Negative	Negative
125548	Mixed/Integrated	Negative	Negative
133858	Mixed/Integrated	Mixed	Integrated
137255	Mixed/Integrated	Mixed	Integrated
139077	Mixed/Integrated	Negative	Negative
139788	Mixed/Integrated	Mixed	Mixed
141013	Mixed/Integrated	Mixed	Integrated
143682	Negative	Negative	Negative
145994	Negative	Negative	Negative
148168	Mixed/Integrated	Mixed	Integrated
148229	Negative	Negative	Negative
149441	Negative	Negative	Negative
150256	Negative	Negative	Negative
152339	Negative	Negative	Negative
154333	Negative	Negative	Negative
155065	Negative	Negative	Negative
156530	Mixed/Integrated	Mixed	Mixed
156548	Negative	Negative	Negative
156775	Negative	Negative	Negative
158876	Negative	Negative	Negative
160072	Mixed/Integrated	Integrated	Integrated
160826	Mixed/Integrated	Negative	Negative
165166	Mixed/Integrated	Integrated	Integrated
166328	Negative	Negative	Negative
168654	Mixed/Integrated	Integrated	Integrated
174170	Negative	Negative	Negative
174703	Mixed/Integrated	Negative	Negative
174820	Mixed/Integrated	Integrated	Integrated
177356	Negative	Negative	Negative
180223	Mixed/Integrated	Integrated	Integrated
181369	Negative	Negative	Negative
184650	Mixed/Integrated	Integrated	Integrated
188937	Mixed/Integrated	Mixed	Mixed
193510	Mixed/Integrated	Integrated	Integrated
194806	Negative	Negative	Negative
197282	Mixed/Integrated	Negative	Negative
204686	Mixed/Integrated	Negative	Integrated
207021	Negative	Negative	Negative
208414	Negative	Negative	Negative
210885	Mixed/Integrated	Integrated	Integrated
211094	Mixed/Integrated	Mixed	Integrated

212134	Negative	Negative	Negative
212834	Negative	Negative	Negative
213545	Mixed/Integrated	Mixed	Integrated
214615	Negative	Negative	Negative
215888	Negative	Negative	Negative
217086	Negative	Negative	Negative
220954	Negative	Negative	Negative
222400	Negative	Negative	Negative
225944	Negative	Negative	Negative
227812	Negative	Negative	Negative
231259	Negative	Negative	Negative
240117	Negative	Negative	Negative
247157	Mixed/Integrated	Negative	Negative
256299	Mixed/Integrated	Integrated	Integrated
256625	Mixed/Integrated	Mixed	Mixed
259668	Mixed/Integrated	Integrated	Integrated
261370	Negative	Negative	Negative
277855	Negative	Negative	Negative
277858	Mixed/Integrated	Mixed	Mixed
282817	Negative	Negative	Negative
282818	Mixed/Integrated	Mixed	Mixed
282820	Mixed/Integrated	Mixed	Integrated
291223	Negative	Negative	Negative
312148	Mixed/Integrated	Mixed	Integrated
325603	Negative	Negative	Negative
349364	Mixed/Integrated	Mixed	Mixed
353495	Negative	Negative	Negative
488306	Negative	Negative	Negative
665595	Negative	Negative	Negative
680063	Negative	Negative	Negative
735515	Negative	Negative	Negative

Table S12. Detection results of DIPS, E2/E6E7 ratio analysis and multiple E1-L1/E6E7 ratio analysis in the HPV 33-positive cases.

Patient No.	DIPS	E2/E6E7 ratio analysis	E1-L1/E6E7 ratio analysis
212306	Negative	Negative	Negative
138664	Negative	Negative	Negative
100835	Negative	Negative	Negative
102127	Negative	Negative	Negative
104022	Negative	Negative	Negative
108976	Mixed/Integrated	Mixed	Integrated
109966	Mixed/Integrated	Mixed	Integrated
112283	Negative	Negative	Negative
117976	Mixed/Integrated	Mixed	Mixed
118710	Mixed/Integrated	Mixed	Integrated
122305	Negative	Negative	Negative
123583	Mixed/Integrated	Mixed	Integrated
123791	Negative	Negative	Negative

124041	Negative	Negative	Negative
124652	Negative	Negative	Negative
130889	Mixed/Integrated	Mixed	Integrated
132410	Negative	Negative	Negative
132740	Negative	Negative	Negative
132903	Negative	Negative	Negative
138697	Negative	Negative	Negative
142675	Mixed/Integrated	Mixed	Integrated
144326	Mixed/Integrated	Mixed	Integrated
145271	Mixed/Integrated	Mixed	Mixed
147910	Negative	Negative	Negative
153355	Negative	Negative	Negative
155711	Negative	Negative	Negative
155712	Negative	Negative	Negative
158070	Negative	Negative	Negative
160125	Mixed/Integrated	Mixed	Integrated
161775	Mixed/Integrated	Mixed	Integrated
161822	Mixed/Integrated	Negative	Integrated
168378	Negative	Negative	Negative
168952	Negative	Negative	Negative
169277	Negative	Negative	Negative
172194	Mixed/Integrated	Integrated	Integrated
172903	Mixed/Integrated	Mixed	Integrated
173326	Mixed/Integrated	Mixed	Integrated
178558	Mixed/Integrated	Negative	Integrated
179379	Negative	Negative	Negative
184219	Negative	Negative	Negative
184770	Negative	Negative	Negative
185456	Negative	Negative	Negative
185629	Mixed/Integrated	Mixed	Integrated
185870	Negative	Negative	Negative
190110	Mixed/Integrated	Mixed	Integrated
190382	Mixed/Integrated	Mixed	Integrated
191052	Mixed/Integrated	Mixed	Integrated
191971	Negative	Negative	Negative
194128	Negative	Negative	Negative
195971	Mixed/Integrated	Mixed	Integrated
197049	Negative	Negative	Negative
197092	Negative	Negative	Negative
197589	Mixed/Integrated	Mixed	Mixed
210352	Mixed/Integrated	Integrated	Integrated
217557	Mixed/Integrated	Negative	Integrated
219024	Mixed/Integrated	Negative	Integrated
219755	Negative	Negative	Negative
219771	Negative	Negative	Negative
220122	Negative	Negative	Negative
222602	Mixed/Integrated	Negative	Integrated
231067	Mixed/Integrated	Integrated	Integrated
232354	Negative	Negative	Negative

233833	Negative	Integrated	Integrated
245045	Negative	Negative	Negative
265740	Mixed/Integrated	Integrated	Integrated
275760	Negative	Negative	Negative
278229	Negative	Negative	Negative
282235	Mixed/Integrated	Integrated	Integrated
290756	Negative	Negative	Negative
290992	Negative	Negative	Negative
294784	Negative	Negative	Negative
295778	Mixed/Integrated	Mixed	Integrated
295949	Negative	Negative	Negative
312258	Negative	Negative	Negative
318024	Negative	Negative	Negative
327629	Negative	Negative	Negative
344399	Mixed/Integrated	Mixed	Mixed
391456	Negative	Negative	Negative
414672	Mixed/Integrated	Mixed	Mixed
460664	Mixed/Integrated	Integrated	Integrated
467264	Mixed/Integrated	Mixed	Mixed
498065	Negative	Negative	Negative
599268	Mixed/Integrated	Mixed	Mixed
600709	Negative	Negative	Negative
617049	Negative	Negative	Negative
636301	Negative	Negative	Negative
701363	Mixed/Integrated	Mixed	Mixed
819486	Negative	Negative	Negative
902724	Negative	Negative	Negative

Table S13. Detection results of DIPS, E2/E6E7 ratio analysis and multiple E1-L1/E6E7 ratio analysis in the HPV 35-positive cases.

Patient No.	DIPS	E2/E6E7 ratio analysis	E1-L1/E6E7 ratio analysis
157835	Negative	Negative	Negative
208488	Negative	Negative	Negative
102988	Negative	Integrated	Integrated
154079	Mixed/Integrated	Mixed	Mixed
155478	Mixed/Integrated	Mixed	Mixed
159571	Negative	Negative	Negative
160446	Negative	Negative	Negative
160739	Mixed/Integrated	Integrated	Integrated
176356	Negative	Integrated	Integrated
189906	Negative	Negative	Negative
223208	Mixed/Integrated	Negative	Integrated
224400	Mixed/Integrated	Mixed	Mixed
276198	Negative	Negative	Negative
285333	Negative	Negative	Negative
286236	Mixed/Integrated	Negative	Integrated
287317	Negative	Negative	Negative
295350	Negative	Negative	Negative

299672	Negative	Negative	Negative
300908	Negative	Negative	Negative
302837	Mixed/Integrated	Mixed	Mixed
365858	Negative	Negative	Negative
372861	Mixed/Integrated	Negative	Integrated
377288	Negative	Negative	Negative
384701	Negative	Negative	Negative
390204	Negative	Negative	Negative
391290	Negative	Negative	Negative
408586	Negative	Negative	Negative
428846	Mixed/Integrated	Mixed	Mixed
764866	Mixed/Integrated	Integrated	Integrated

Table S14. Detection results of DIPS, E2/E6E7 ratio analysis and multiple E1-L1/E6E7 ratio analysis in the HPV 39-positive cases.

Patient No.	DIPS	E2/E6E7 ratio analysis	E1-L1/E6E7 ratio analysis
136573	Negative	Negative	Negative
185076	Negative	Negative	Negative
106876	Mixed/Integrated	Integrated	Integrated
109830	Mixed/Integrated	Mixed	Mixed
115641	Negative	Negative	Negative
142148	Mixed/Integrated	Mixed	Mixed
142164	Negative	Negative	Negative
146202	Negative	Negative	Negative
147429	Negative	Negative	Negative
154672	Negative	Negative	Negative
181735	Mixed/Integrated	Negative	Integrated
183437	Mixed/Integrated	Mixed	Mixed
184062	Negative	Negative	Negative
186750	Mixed/Integrated	Integrated	Integrated
194591	Mixed/Integrated	Mixed	Mixed
196599	Negative	Negative	Negative
201392	Mixed/Integrated	Mixed	Mixed
207620	Negative	Negative	Negative
212177	Mixed/Integrated	Integrated	Integrated
216233	Mixed/Integrated	Mixed	Mixed
263063	Negative	Negative	Negative
319670	Negative	Negative	Negative
319670	Negative	Negative	Negative
444704	Negative	Negative	Negative
483542	Negative	Negative	Negative
494628	Negative	Negative	Negative
501405	Mixed/Integrated	Negative	Integrated
513723	Mixed/Integrated	Mixed	Mixed
611626	Negative	Negative	Negative
720102	Negative	Negative	Negative
729465	Negative	Negative	Negative
752867	Negative	Negative	Negative

775489	Negative	Negative	Negative
910816	Mixed/Integrated	Negative	Integrated
983949	Mixed/Integrated	Mixed	Mixed

Table S15. Detection results of DIPS, E2/E6E7 ratio analysis and multiple E1-L1/E6E7 ratio analysis in the HPV 41-positive cases.

Patient No.	DIPS	E2/E6E7 ratio analysis	E1-L1/E6E7 ratio analysis
189250	Negative	Negative	Negative
273781	Negative	Negative	Negative
195880	Negative	Negative	Negative

Table S16. Detection results of DIPS, E2/E6E7 ratio analysis and multiple E1-L1/E6E7 ratio analysis in the HPV 42-positive cases.

Patient No.	DIPS	E2/E6E7 ratio analysis	E1-L1/E6E7 ratio analysis
179630	Negative	Negative	Negative
576561	Negative	Negative	Negative
215633	Negative	Negative	Negative
226983	Mixed/Integrated	Negative	Negative
236801	Negative	Negative	Negative

Table S17. Detection results of DIPS, E2/E6E7 ratio analysis and multiple E1-L1/E6E7 ratio analysis in the HPV 45-positive cases.

Patient No.	DIPS	E2/E6E7 ratio analysis	E1-L1/E6E7 ratio analysis
398345	Negative	Negative	Negative
102706	Negative	Negative	Negative
180759	Mixed/Integrated	Mixed	Mixed
186083	Mixed/Integrated	Mixed	Mixed
507109	Mixed/Integrated	Mixed	Mixed
612676	Negative	Negative	Negative
718312	Mixed/Integrated	Mixed	Mixed
806524	Negative	Negative	Negative

Table S18. Detection results of DIPS, E2/E6E7 ratio analysis and multiple E1-L1/E6E7 ratio analysis in the HPV 51-positive cases.

Patient No.	DIPS	E2/E6E7 ratio analysis	E1-L1/E6E7 ratio analysis
104450	Negative	Negative	Negative
130826	Negative	Negative	Negative
113429	Negative	Negative	Negative
158660	Mixed/Integrated	Mixed	Mixed
258020	Mixed/Integrated	Integrated	Integrated
283920	Negative	Negative	Negative
343990	Mixed/Integrated	Mixed	Mixed
684152	Negative	Negative	Negative

Table S19. Detection results of DIPS, E2/E6E7 ratio analysis and multiple E1-L1/E6E7 ratio analysis in the HPV 52-positive cases.

Patient No.	DIPS	E2/E6E7 ratio analysis	E1-L1/E6E7 ratio analysis
174862	Negative	Negative	Negative
237233	Negative	Negative	Negative
105274	Mixed/Integrated	Mixed	Mixed
142149	Negative	Negative	Negative
142474	Negative	Negative	Negative
146280	Mixed/Integrated	Negative	Negative
156899	Mixed/Integrated	Negative	Negative
167239	Mixed/Integrated	Integrated	Integrated
224500	Negative	Negative	Negative
236501	Negative	Negative	Negative
331300	Negative	Negative	Negative
455137	Negative	Negative	Negative
466036	Mixed/Integrated	Mixed	Integrated

Table S20. Detection results of DIPS, E2/E6E7 ratio analysis and multiple E1-L1/E6E7 ratio analysis in the HPV 53-positive cases.

Patient No.	DIPS	E2/E6E7 ratio analysis	E1-L1/E6E7 ratio analysis
108544	Negative	Negative	Negative
528778	Negative	Negative	Negative
132080	Negative	Negative	Negative
143224	Negative	Negative	Negative
147282	Mixed/Integrated	Negative	Negative
152583	Negative	Negative	Negative
160958	Mixed/Integrated	Negative	Negative
252533	Negative	Negative	Negative
262455	Negative	Negative	Negative
270406	Mixed/Integrated	Integrated	Integrated
273813	Negative	Negative	Negative
281962	Negative	Negative	Negative
285171	Negative	Negative	Negative
288199	Negative	Negative	Negative
289715	Negative	Negative	Negative
296530	Negative	Negative	Negative
307888	Negative	Negative	Negative
356276	Negative	Negative	Negative
409836	Mixed/Integrated	Mixed	Mixed
442858	Negative	Negative	Negative
445398	Negative	Negative	Negative
467319	Negative	Negative	Negative
486398	Mixed/Integrated	Integrated	Integrated
497861	Mixed/Integrated	Mixed	Integrated
528801	Negative	Negative	Negative

646062	Negative	Negative	Negative

Table S21. Detection results of DIPS, E2/E6E7 ratio analysis and multiple E1-L1/E6E7 ratio analysis in the HPV 56-positive cases.

Patient No.	DIPS	E2/E6E7 ratio analysis	E1-L1/E6E7 ratio analysis
472120	Negative	Negative	Negative
492121	Negative	Negative	Negative
171760	Negative	Negative	Negative
211503	Mixed/Integrated	Mixed	Mixed
289221	Negative	Negative	Negative
294827	Negative	Negative	Negative
304508	Negative	Negative	Negative
309537	Mixed/Integrated	Integrated	Integrated
319663	Negative	Negative	Negative
351797	Mixed/Integrated	Mixed	Mixed
361717	Mixed/Integrated	Integrated	Integrated
444280	Negative	Negative	Negative
447802	Negative	Negative	Negative
476196	Mixed/Integrated	Mixed	Mixed
564327	Negative	Negative	Negative
568528	Negative	Negative	Negative
576928	Negative	Negative	Negative
823340	Negative	Negative	Negative
846461	Negative	Negative	Negative
850723	Negative	Negative	Negative
960540	Negative	Negative	Negative

Table S22. Detection results of DIPS, E2/E6E7 ratio analysis and multiple E1-L1/E6E7 ratio analysis in the HPV 58-positive cases.

Patient No.	DIPS	E2/E6E7 ratio analysis	E1-L1/E6E7 ratio analysis
389880	Negative	Negative	Negative
282523	Negative	Negative	Negative
100241	Negative	Negative	Negative
142951	Negative	Negative	Negative
143313	Negative	Negative	Negative
269139	Negative	Negative	Negative
279976	Negative	Negative	Negative
312650	Negative	Negative	Negative
316063	Negative	Negative	Negative
319475	Negative	Negative	Negative
340970	Negative	Negative	Negative
399486	Mixed/Integrated	Negative	Mixed
478612	Negative	Negative	Negative
493119	Negative	Negative	Negative
522897	Mixed/Integrated	Integrated	Integrated
645642	Negative	Negative	Negative

649737	Negative	Negative	Negative
779644	Mixed/Integrated	Mixed	Mixed
988869	Negative	Negative	Negative

Table S23. Detection results of DIPS, E2/E6E7 ratio analysis and multiple E1-L1/E6E7 ratio analysis in the HPV 59-positive cases.

Patient No.	DIPS	E2/E6E7 ratio analysis	E1-L1/E6E7 ratio analysis
161077	Negative	Negative	Negative
135658	Negative	Negative	Negative
101740	Mixed/Integrated	Mixed	Mixed
343726	Negative	Negative	Negative
349974	Negative	Negative	Negative
554048	Negative	Negative	Negative
904231	Negative	Negative	Negative

Table S24. Detection results of DIPS, E2/E6E7 ratio analysis and multiple E1-L1/E6E7 ratio analysis in the HPV 61-positive cases.

Patient No.	DIPS	E2/E6E7 ratio analysis	E1-L1/E6E7 ratio analysis
396143	Negative	Negative	Negative
141277	Negative	Negative	Negative
377376	Mixed/Integrated	Mixed	Mixed
510643	Negative	Negative	Negative
754323	Negative	Negative	Negative

Table S25. Detection results of DIPS, E2/E6E7 ratio analysis and multiple E1-L1/E6E7 ratio analysis in the HPV 62-positive cases.

Patient No.	DIPS	E2/E6E7 ratio analysis	E1-L1/E6E7 ratio analysis
522687	Negative	Negative	Negative
364480	Negative	Negative	Negative
345404	Mixed/Integrated	Negative	Integrated
432087	Negative	Negative	Negative
436221	Negative	Negative	Negative
624287	Negative	Mixed	Mixed

Table S26. Detection results of DIPS, E2/E6E7 ratio analysis and multiple E1-L1/E6E7 ratio analysis in the HPV 66-positive cases.

Patient No.	DIPS	E2/E6E7 ratio analysis	E1-L1/E6E7 ratio analysis
445147	Negative	Negative	Negative
781823	Negative	Negative	Negative
214843	Negative	Negative	Negative
218320	Negative	Negative	Negative
265763	Mixed/Integrated	Mixed	Mixed
282271	Mixed/Integrated	Integrated	Integrated

332315	Mixed/Integrated	Mixed	Mixed
332610	Negative	Negative	Negative
338511	Mixed/Integrated	Mixed	Mixed
347462	Negative	Negative	Negative
349112	Negative	Negative	Negative
359842	Negative	Negative	Negative
368090	Negative	Negative	Negative
416685	Negative	Negative	Negative
792138	Negative	Negative	Negative
808639	Negative	Negative	Negative

Table S27. Detection results of DIPS, E2/E6E7 ratio analysis and multiple E1-L1/E6E7 ratio analysis in the HPV 68-positive cases.

Patient No.	DIPS	E2/E6E7 ratio analysis	E1-L1/E6E7 ratio analysis
345771	Negative	Negative	Negative
344005	Negative	Negative	Negative
149708	Negative	Negative	Negative
162073	Negative	Negative	Negative
166310	Negative	Negative	Negative
214310	Mixed/Integrated	Negative	Integrated
293474	Negative	Negative	Negative
294364	Negative	Negative	Negative
306731	Mixed/Integrated	Integrated	Integrated
329086	Negative	Negative	Negative
499939	Negative	Negative	Negative
562428	Negative	Negative	Negative
573266	Mixed/Integrated	Mixed	Integrated
667985	Mixed/Integrated	Mixed	Mixed
805513	Negative	Negative	Negative
813214	Negative	Negative	Negative
839515	Negative	Negative	Negative
873927	Negative	Negative	Negative

Table S28. Detection results of DIPS, E2/E6E7 ratio analysis and multiple E1-L1/E6E7 ratio analysis in the HPV 69-positive cases.

Patient No.	DIPS	E2/E6E7 ratio analysis	E1-L1/E6E7 ratio analysis
172271	Negative	Negative	Negative
358077	Negative	Negative	Negative
111441	Negative	Negative	Negative
297880	Mixed/Integrated	Mixed	Integrated
383298	Negative	Negative	Negative
416993	Mixed/Integrated	Integrated	Integrated
745366	Negative	Negative	Negative

Table S29. Detection results of DIPS, E2/E6E7 ratio analysis and multiple E1-L1/E6E7 ratio analysis in the HPV 74-positive cases.

Patient No.	DIPS	E2/E6E7 ratio analysis	E1-L1/E6E7 ratio analysis
174040	Negative	Negative	Negative

Table S30. Detection results of DIPS, E2/E6E7 ratio analysis and multiple E1-L1/E6E7 ratio analysis in the HPV 93-positive cases.

Patient No.	DIPS	E2/E6E7 ratio analysis	E1-L1/E6E7 ratio analysis
582333	Negative	Negative	Negative
