# Supplementary material for: Diagnosis of 25 genotypes of human papillomaviruses for their physical statuses in cervical precancerous/cancerous lesions: a comparison of E2/E6E7 ratio-based vs. multiple E1-L1/E6E7 ratio-based detection techniques
Source: J Transl Med. 2014 Oct 2;12:282. doi: 10.1186/s12967-014-0282-2 (PMC4192431; doi:10.1186/s12967-014-0282-2)
Supplement: Additional file 2: — Supplementary Figures. Figure S1 schematically depicts the principal of DIPS and provides some sequencing-verified samples of the E2-related and non-E2 HPV integration events. In addition, an example of the undetected cases of DIPS, which was corrected by changing a restriction enzyme, is displayed. Figure S2 shows the age-, genotype- and cervical lesion-related distribution patterns of the integrated/mixed HPV infection that was detected by the three techniques among the enrolled women. Figure S3 describes the distribution characteristics of the DIPS-, E2/E6E7 ratio analysis- and E1-L1/E6E7 ratio analysis-detected cases among the viral load-cervical lesion two-dimensional space, where the y-axis consists of three DNA load tertiles, and the x-axis consists of five biopsy-identified lesion grades. [file 12967_2014_282_MOESM2_ESM.pdf]

Supplementary figures

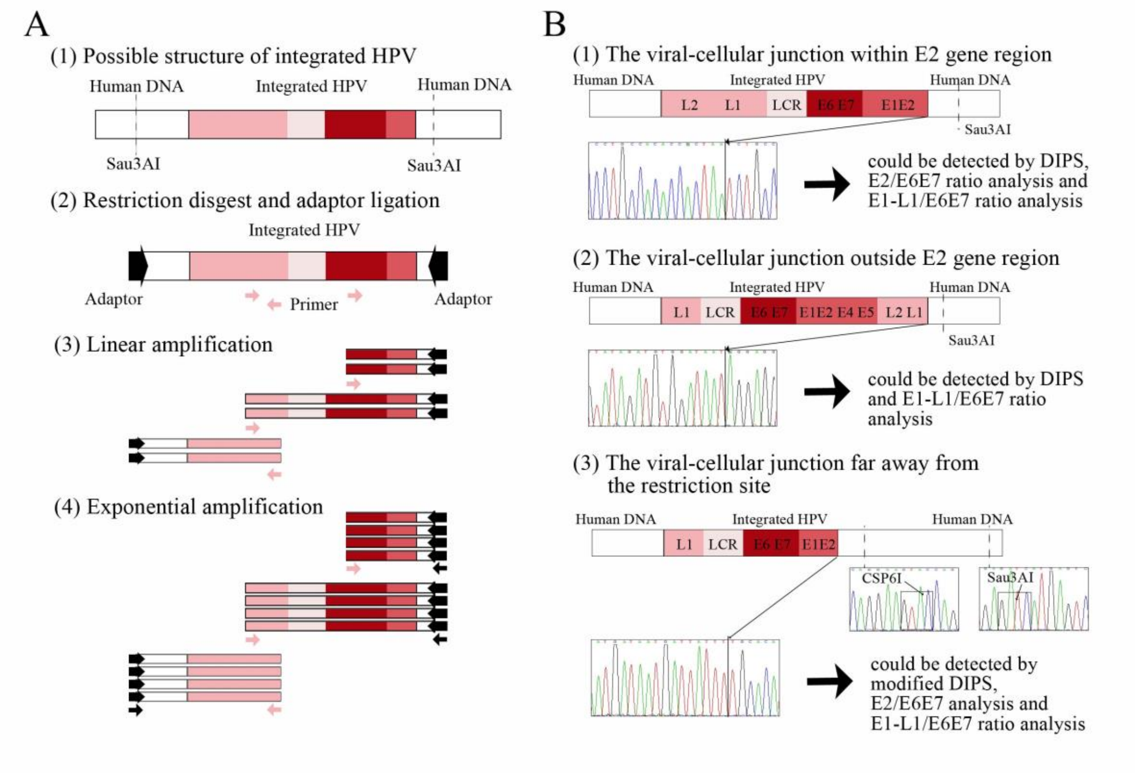

Figure S1. Schematic representation of the operating principal of DIPS and some typical examples of the detection results.

A. The workflow of DIPS is given. Initially, the cellular DNA was digested with a restriction enzyme, and the obtained fragments were ligated with a restriction-site-specific adaptor. Then, the viral-cellular-adaptor sequences were linearly amplified using HPV-specific primers. This step ensured that the viral-cellular junction-containing sequences could be selectively amplified before an exponential amplification process. Finally, the viral-cellular chimeric sequences were amplified with a standard PCR method. The products were subjected to DNA sequencing, and the viral-cellular junctions were identified. **B.** DIPS detects the viral-cellular junctions that are located within or outside E2 gene region. However, if a restriction site is far from the viral-cellular junction, the junction-containing sequence cannot be amplified by standard PCR; false-negative results, therefore, would be produced. In this study, a case (Patient No. 189543) that was false-negatively diagnosed by DIPS was corrected by changing the restriction enzymes Sau3AI and TaqI to CSP6I.

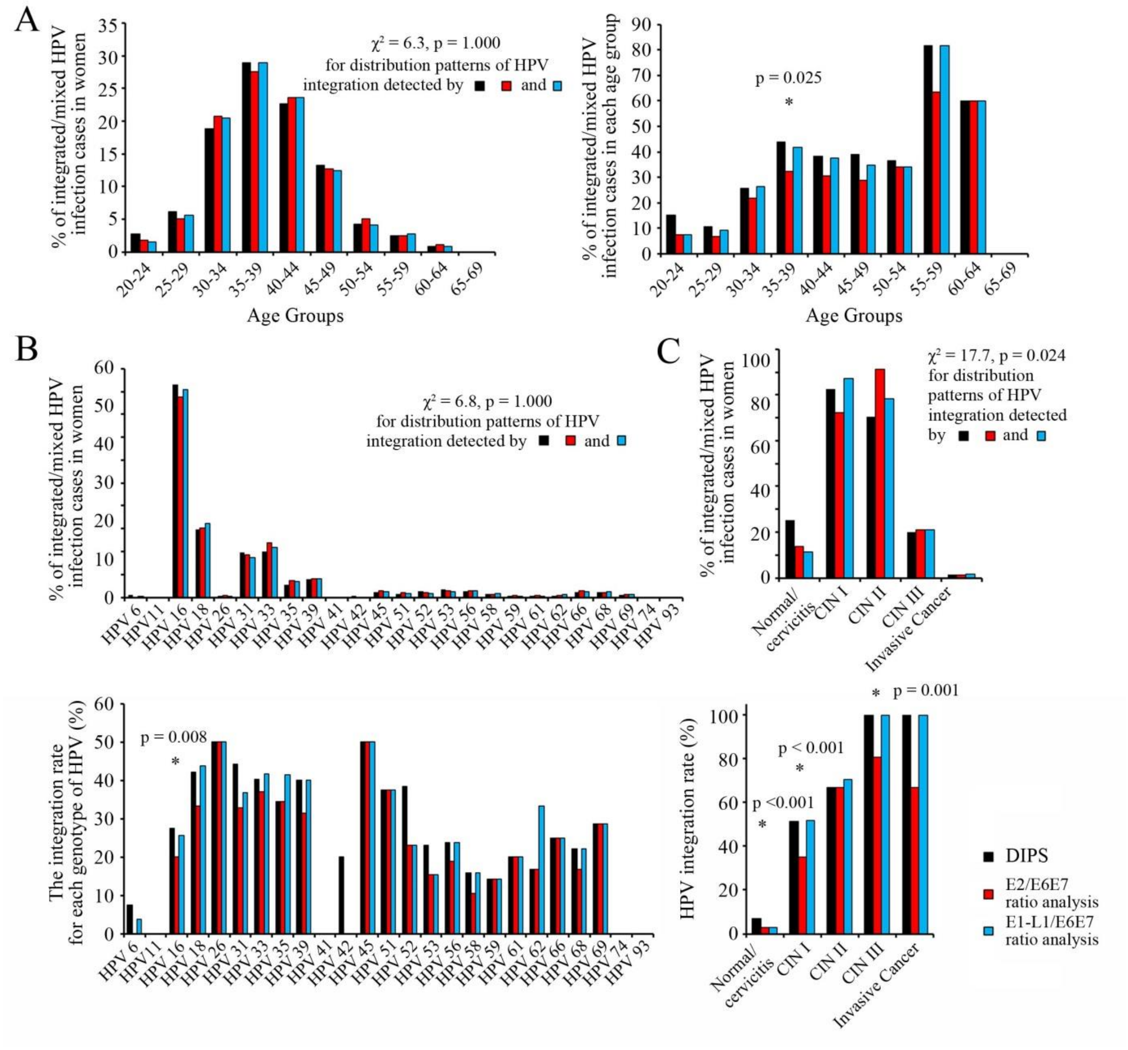

Figure S2. Age-, genotype- and cervical-lesion-related distribution patterns of HPV integration.

A. The percentages of integrated/mixed HPV infection of each age group among the enrolled women (left) and the percentages of integrated/mixed HPV infection within each age group (right). **B.** The percentages of integrated/mixed HPV infection of each viral genotype among the enrolled women (upper) and the percentages of integrated/mixed HPV infection within each viral genotype (lower). **C.** The percentages of integrated/mixed HPV infection of each cervical lesion grade among the enrolled women (upper) and the percentages of integrated/mixed HPV infection within each cervical lesion grade (lower). A two-sided χ^2 test was used to evaluate the differences between the distribution patterns of the three techniques. For percentage variations concerning all of the enrolled women, a two-sided χ^2 test was used to analyze the differences between the distribution modes that were caused by the three techniques. For percentage variations concerning a specific group of women, such as those of a certain age, with a certain genotype of HPV infection and with a certain cervical lesion grade, a two-sided χ^2 test was used to compare the differences in the HPV integration rates that were caused by the three techniques only within this group. *, statistical significance.

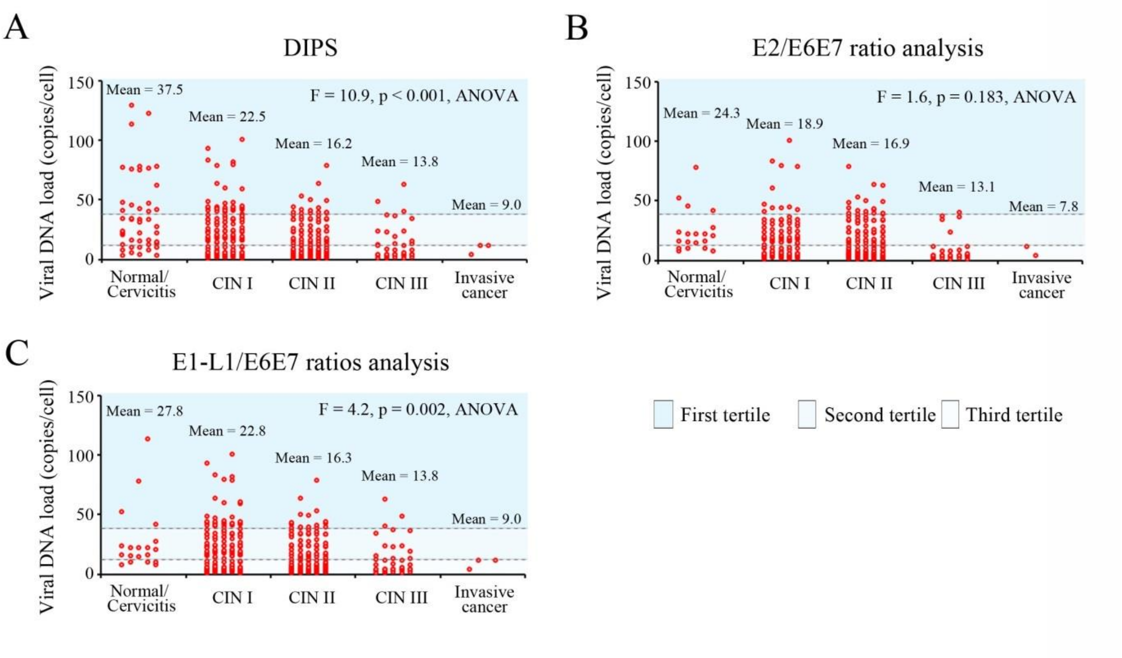

Figure S3. The distribution characteristics of the DIPS-, E2/E6E7-ratio-analysis- and the E1-L1/E6E7-ratio-analysis-detected cases.

A. The viral load-cervical lesion distribution characteristics of the DIPS-detected integrated/mixed HPV infection. **B.** The viral load-cervical lesion distribution characteristics of the E2/E6E7 ratio analysis-detected integrated/mixed HPV infection. **C.** The viral load-cervical lesion distribution characteristics of the E1-L1 ratio analysis-detected integrated/mixed HPV infection. An ANOVA was used to compare the means of the viral DNA loads between different grades of cervical lesions. The viral loads were divided into tertiles, which were, from high to low, named as the first, second and third tertiles, respectively.
